# Oxytocin Facilitates Allomaternal Behavior under Stress in Laboratory Mice

**DOI:** 10.1523/ENEURO.0405-21.2022

**Published:** 2022-02-17

**Authors:** Yousuke Tsuneoka, Chihiro Yoshihara, Ryuko Ohnishi, Sachine Yoshida, Eri Miyazawa, Masanobu Yamada, Kazuhiko Horiguchi, W. Scott Young, Katsuhiko Nishimori, Tadafumi Kato, Kumi O. Kuroda

**Affiliations:** 1 Laboratory for Affiliative Social Behavior, RIKEN Center for Brain Science, Wako 351-0198, Japan; 2 Department of Internal Medicine, Division of Endocrinology and Metabolism, Gunma University Graduate School of Medicine, Maebashi 371-8511, Japan; 3 Section on Neural Gene Expression, National Institute of Mental Health, National Institutes of Health, Bethesda, MD 20892-9663; 4 Department of Obesity and Internal Inflammation, Fukushima Medical University, Fukushima 960-1295, Japan; 5 Department of Psychiatry and Behavioral Science, Graduate School of Medicine, Juntendo University, Tokyo 113-0033, Japan

**Keywords:** maternal behavior, medial preoptic area, *Mus musculus*, oxytocin, vasopressin

## Abstract

Oxytocin (Oxt) controls reproductive physiology and various kinds of social behaviors, but the exact contribution of Oxt to different components of parental care still needs to be determined. Here, we illustrate the neuroanatomical relations of the parental nurturing-induced neuronal activation with magnocellular Oxt neurons and fibers in the medial preoptic area (MPOA), the brain region critical for parental and alloparental behaviors. We used genetically-targeted mouse lines for *Oxt*, *Oxt receptor* (*Oxtr*), *vasopressin receptor 1a* (*Avpr1a*), *vasopressin receptor 1b* (*Avpr1b*), and *thyrotropin-releasing hormone* (*Trh*) to systematically examine the role of Oxt-related signaling in pup-directed behaviors. The *Oxtr*-*Avpr1a*-*Avpr1b* triple knock-out (TKO), and *Oxt*-*Trh*-*Avpr1a*-*Avpr1b* quadruple KO (QKO) mice were grossly healthy and fertile, except for their complete deficiency in milk ejection and modest deficiency in parturition secondary to maternal loss of the *Oxt* or *Oxtr* gene. In our minimal stress conditions, pup-directed behaviors in TKO and QKO mothers and fathers, virgin females and males were essentially indistinguishable from those of their littermates with other genotypes. However, *Oxtr* KO virgin females did show decreased pup retrieval in the pup-exposure assay performed right after restraint stress. This stress vulnerability in the *Oxtr* KO was abolished by the additional *Avpr1b* KO. The general stress sensitivity, as measured by plasma cortisol elevation after restraint stress or by the behavioral performance in the open field (OF) and elevated plus maze (EPM), were not altered in the *Oxtr* KO but were reduced in the *Avpr1b* KO females, indicating that the balance of neurohypophysial hormones affects the outcome of pup-directed behaviors.

## Significance Statement

Parental care without suckling induces the most significant transcriptional activation in the caregivers’ anterior commissural nucleus (AC), the third-largest population of oxytocin (Oxt) neurons in the medial preoptic area (MPOA), but not in its nonoxytocinergic neurons. The pup-directed behaviors in postpartum mothers, fathers and virgin males and females of *Oxt receptor* (*Oxtr*), *vasopressin receptor* (*Avpr*) *1a* and *1b* triple knock-out (TKO) mice were essentially normal in our standard experimental conditions with minimal stress. Under stressful conditions, however, *Oxtr* KO mice showed decreased parental nurturing behaviors, which was compensated for by combining with the *Avpr1b* KO.

## Introduction

Oxytocin (Oxt) and vasopressin (Avp) are nonapeptide hormones with a common ancestor gene. Avp is critically involved in osmoregulation and Oxt stimulates uterine contraction during parturition and milk-ejection during nursing (Robinson and Verbalis, 2003; Wakerley, 2005). In addition to these peripheral functions, Oxt and Avp have direct actions onto neurons expressing their receptors and participate in the control of anxiety, pain, and stress responses, social recognition, pair bonding, and aggression (Insel, 2010; Neumann and Landgraf, 2012; Onaka et al., 2012; Hurlemann and Grinevich, 2017; Grinevich and Neumann, 2021).

For maternal and allomaternal behaviors, there are numerous reports supporting the positive effects of Oxt (Takayanagi et al., 2005; Caldwell and Young, 2006; Marlin et al., 2015). The facilitatory roles of Oxt in parental care are reported most frequently during the high-stress conditions, such as the onset/initial learning phase of parenting or in a nonhome cage environment (Pedersen et al., 1982; Marlin et al., 2015; Carcea et al., 2021). In these cases, however, Oxt facilitation of maternal behavior could be because of Oxt’s anxiolytic/anti-stress effect in general, rather than via its primary role in maternal behavior *per se* (McCarthy et al., 1992; McCarthy, 1995; Yoshihara et al., 2017). Moreover, there are several studies reporting that multiple components of parental behavior are intact in mice harboring genetic mutations of the Oxt-Oxytocin receptor (Oxtr) system (Nishimori et al., 1996; Young et al., 1996; Gross et al., 1998; Macbeth et al., 2010), impeding a coherent explanation of the exact role of Oxt in different components of parental care. One of the confounding factors is the possible cross-activation/compensatory mechanisms of the Oxt and Avp systems via their receptors. Specifically, >80% structural homology between Oxtr and Avp receptor 1a (Avpr1a) causes significant cross-activation (Manning et al., 2012; Jurek and Neumann, 2018), which is not negligible as both Oxt and Avp are implicated in maternal behavior (Bosch and Neumann, 2008).

**Figure 1.**
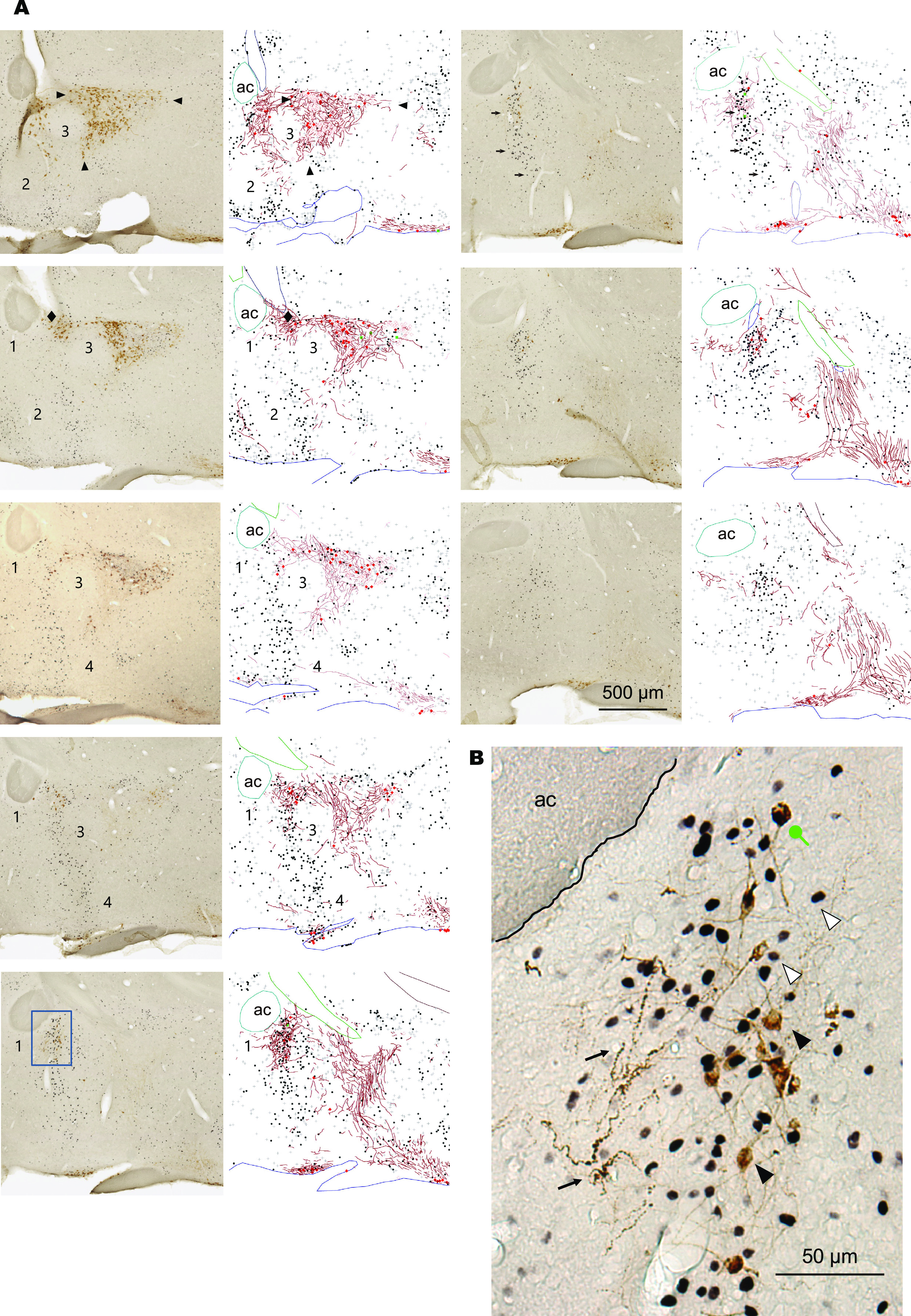
Sagittal view of anatomic distribution of Oxt neurons, fibers, and parenting-induced c-Fos expression. Distribution of NPI (brown)-ir and c-Fos (black)-ir cell in and around of MPOA of virgin females after pup exposure (parasagittal section). ***A***, left and right panels, Representative photographs and their diagrammatic drawings, respectively. These sections were stained by IHC. Black squares and plus (+) symbols, respectively, represent strongly and weakly expressed c-Fos-ir neurons without NPI-ir. Filled and open red circles, respectively, represent strongly and weakly NPI-ir cell bodies without c-Fos signals. Green squares, respectively, represent NPI-ir cell bodies with c-Fos-ir. Red lines represent NPI-ir fibers. Numbers 1–4 show the areas relatively devoid of c-Fos-ir, NPI-ir neurons, and fibers. Arrows indicate Oxt-ir thick dendrites with a corkscrew-like morphology and irregular varicosities. ac, anterior commissure. Panels are arranged in the medial–lateral order, from the left top to left bottom, and the right top to right bottom. ***B***, High-magnification image of the blue squared region in ***A***, containing the AC. The circle-headed arrow indicates double-labeled cells of NPI and c-Fos. White arrowheads indicate single-labeled cells of c-Fos, black arrowheads indicate single-labeled cells of NPI, and small arrows indicate NPI-ir fibers.

**Figure 2.**
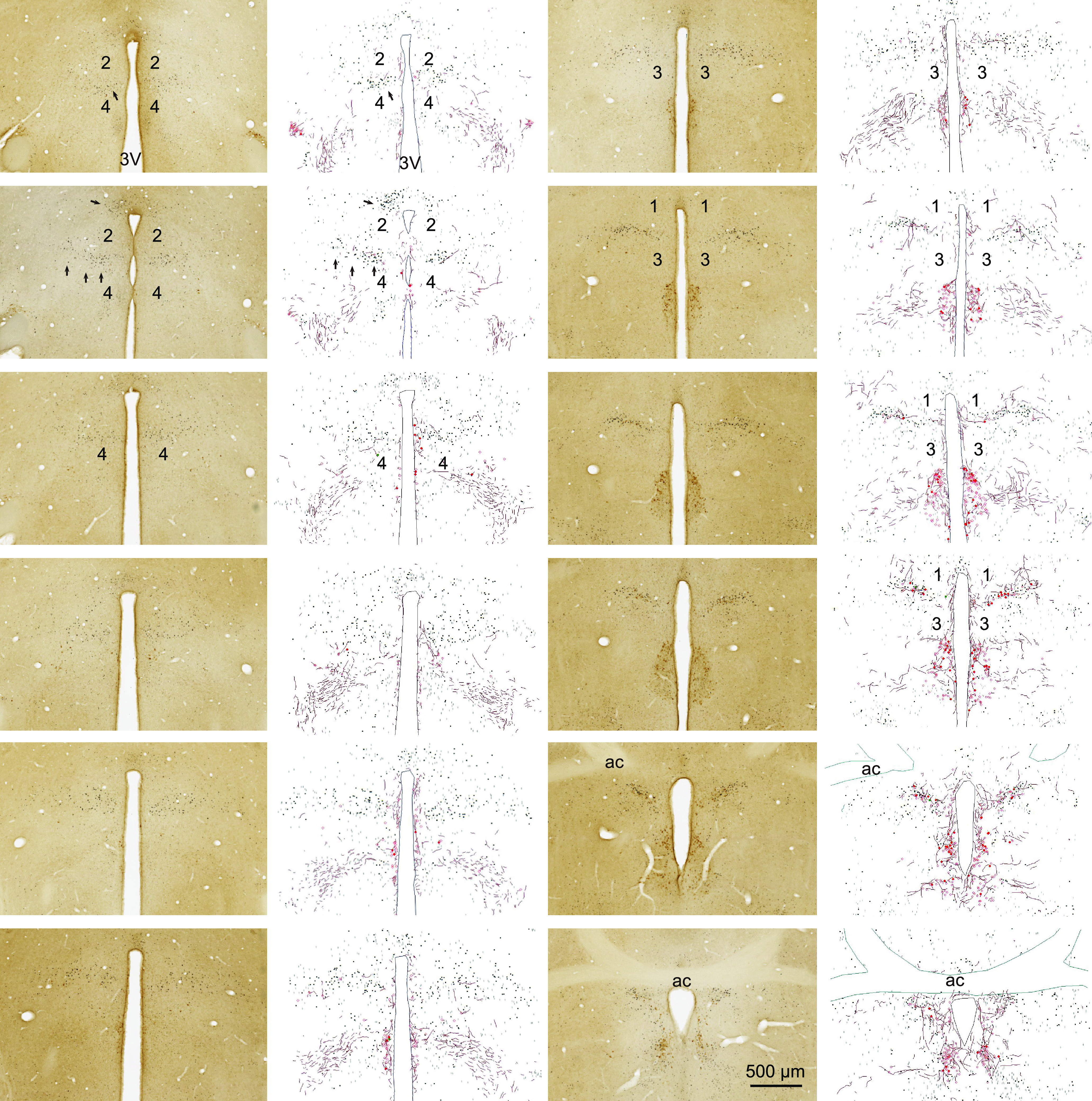
Horizontal view of anatomic distribution of Oxt neurons, fibers, and parenting-induced c-Fos expression. Distribution of NPI (brown)-ir and c-Fos (black)-ir cell in and around of MPOA of virgin females after pup exposure (horizontal section). Left and right panels show representative photographs and their diagrammatic drawings, respectively. These sections were stained by IHC. Black squares and plus symbols, respectively, represent strongly and weakly expressed c-Fos-ir neurons. Filled and open red circles, respectively, represent NPI-ir cell bodies with or without c-Fos signals. Red lines represent NPI-ir fibers. Numbers 1–4 show the areas relatively devoid of c-Fos-ir, NPI-ir neurons, and fibers. 3V, 3rd ventricle; ac, anterior commissure. All panels are arranged in ventral–dorsal order from the left, top to bottom, and then the right, top to bottom.

From the viewpoint of functional neuroanatomy, we have previously studied the pattern of neuronal activation during maternal, paternal and allomaternal care in mice (Tsuneoka et al., 2013, 2015). We focused on the medial preoptic area (MPOA), the critical brain area for parental care (Numan, 1974, 2020; Numan and Numan, 1994), and investigated expression of c-Fos protein, the component of AP-1 transcription factor as a reliable readout of neuronal activation (Herdegen and Leah, 1998). The most pronounced c-Fos expression after 2 h of nurturing behavior is observed at the anterior commissural nucleus (AC; previously abbreviated as ACN) of the MPOA. The AC contains the third largest population of magnocellular Oxt neurons, Oxt fibers as well as Avp fibers, and thyrotropin-releasing hormone (Trh)-producing neurons (Peterson, 1966; Armstrong et al., 1980; Rhodes et al., 1981; Sofroniew, 1985; Castel and Morris, 1988; Grinevich and Akmayev, 1997). Both oxytocinergic and nonoxytocinergic AC neurons are c-Fos positive after 2 h of pup exposure in postpartum mothers. In virgin females, however, the transcriptionally-activated AC neurons during allomaternal behavior are essentially nonoxytocinergic, while roughly 40% of these activated AC neurons expressed calcitonin receptor (Calcr; Yoshihara et al., 2021). Moreover, while Calcr neurons in the central part of the MPOA (cMPOA), which lies ventrally to the AC, are functionally critical for parental nurturing (Yoshihara et al., 2021), all nonoxytocinergic AC neurons, including Calcr neurons, are not. Still, the close spatial correlation of the parenting-induced activation pattern and the distribution of Oxt neurons and fibers in the MPOA (Figs. 1, 2; Tsuneoka et al., 2013) is remarkable, and prompted us to further investigate this issue. Here, to elucidate the exact role of Oxt system in parental care, we used seven lines of genetic mutant mice in combination, covering genes for *Oxt*, *Trh*, *Oxtr*, and *Avpr1a* and *Avpr1b*, and performed a systematic investigation of the role of Oxt-Avp system in postpartum-maternal, postpartum-paternal, and allomaternal behaviors, as well as infanticide of virgin male mice (collectively called “pup-directed behaviors”).

## Materials and Methods

### Animals

All animal experimentation was approved by and conducted in accordance with regulations of the Animal Experiment Judging Committee of RIKEN, which was based on the National Institutes of Health guide *Principles of Laboratory Animal Care* (NIH publication no. 85–23, revised 1985). The male C57BL/6J mice used for this study were obtained originally from The Jackson Laboratory. The oxytocin (B6;129S-*Oxt^tm1Wsy^*/J; stock #2713; Young et al., 1996), *Avpr1a* (B6.129P2-*Avpr1a^tm1Dgen^*/J; stock #5776; Deltagen, Inc. 2005) and *Avpr1b* knock-out (KO) mouse strains (B6;129 × 1-*Avpr1b^tm1Wsy^*/J; stock #6160; Wersinger et al., 2002) were obtained from The Jackson Laboratory. A *Oxtr* KO mouse strain described in (Takayanagi et al., 2005) and a *Trh* KO mouse strain described in (Yamada et al., 1997) were developed by co-authors and available from their laboratories. Another *Avpr1a* and *1b* double KO (DKO) mouse strain (B6;129Sv-*V1a^tm1Gzt^V1b^tm1Gzt^*; stock #559; Tanoue et al., 2004; Koshimizu et al., 2006) was obtained from the Center for Animal Resources and Development, Kumamoto University (Kumamoto, Japan). All the mice, except for the *Avpr1b* KO mouse in the quadruple KO (QKO) study, were backcrossed more than seven times to C57BL/6J mouse. The *Avpr1a* (JAX 5776), *Avpr1b* (JAX 6160), *Oxt* and *Trh* QKO mice line was named QKO; *Avpr1a* (JAX 5776), *Avpr1b* (JAX 6160), and *Oxtr* triple KO (TKO) mice line was named TKO; and *Avpr1a*, *Avpr1b* (*V1a^tm1Gzt^V1b^tm1Gzt^*, Kumamoto University stock #559), and *Oxtr* TKO mice line was named HIR in this study.

Mice were raised in our breeding colony under controlled conditions (12 h light/dark cycle; lights on at 8 A.M.; 23 ± 2°C; 55 ± 10% humidity; and *ad libitum* access to water and food). Mice were weaned at four weeks of age and were housed in groups of four or five in ventilated shoebox cages (267 × 483 × 152 mm) with TEK-Fresh bedding (Harlan). All mice were 12–24 weeks old at the start of experiments.

For generating QKO mice, the *Avpr1b* mouse line were a C57BL/6J × 129 mixed background. For generating TKO mice, we used the *Avpr1b* mouse line which were backcrossed five times to C57BL/6J background. Breeding of genetic mutant mice were performed as described (Kuroda et al., 2011); briefly, first, DKO mice of approximate combinations were produced by crossbreeding of single-KO mice. Then, we crossbred different combination of DKO mice to produce QKO or TKO mice. The TKO male mice were crossbred to triple-heterozygous or single-KO/double-heterozygous female mice, and their littermate offspring were used for the behavioral testing. After the QKO male mice were obtained, they were crossbred to DKO/double-heterozygous or single-KO/triple-heterozygous female mice, and the offspring were used for the behavioral testing.

The genotypes of mice were determined by electrophoresis of the product from polymerase chain reaction using Takara Ex Taq (Takara Bio Inc.) or Quick Taq HS DyeMix (TOYOBO) with the specific primer sets for each mouse strain as described (Table 1). The genomic DNA was obtained from last 2 mm of tail. Samples were put into 100 μl of proteinase K solution [100 mm Tris-HCl (pH 8.0), 5 mm EDTA, 200 mm NaCl, 0.2% (w/v) SDS, 2% (v/v) 15 mg/ml proteinase K (Takara Bio Inc.)] and heated at 56°C for 2 h, 95°C for 2 min, and then diluted by 300-μl distilled water. The genotyping was doubly confirmed after the end of each experiment.

**Table 1:** Primers used for genotyping

Strain	Gene loci	Primer sequence (5′ to 3′)	Tm
B6;129S-*Oxt^tm1Wsy^* /J;	*Oxt*	gtgctggacctggatatgcgcaag	68
		agcgtcctttgccgcccgggccgcaggggagacactgtggctgtgg	
		ctgctaaagcgcatgctccagactgc	
*Trh* KO	*Trh*	tctcgtcgtgacccatggcgatg	64
		tctcgtcgtgacccatggcgatg	
		ttactcctccagaggttccctgac	
B6.129P2-*Avpr1a ^tm1Dgen^* /J	*Avpr 1a*	cgcaacgaggagctggcgaagctgg	64
		gcggtaggtgatgtcccagcacagc	
		gggccagctcattcctcccactcat	
*Oxtr* KO	*Oxtr*	gttgggaacagcggtgatta	64
		ccttggaagcaggaggtgaag	
		gctgcgcagtggtggtgacttc	
B6;129×1-*Avpr1b ^tm1Wsy^* /J	*Avpr 1b*	accccttcccagcctctgagcccagaaagcgaagg	64
		gaaacggctactctctccgattccaaaagaaag	
		acctgtagatatttgacagcccgg	
B6;129Sv-*V1a^tm1Gzt^V1b^tm1Gzt^*	*Avpr 1a*	acaagtgttttgtaactagtgactcta	60
		aggggcttctggtcacgccttgt	
		acatagcgttggctacccgtgat	
	*Avpr 1b*	gaaacggctactctctcgattccaaaagaag	60
		gcgaattcgatatcaagcttatcga	
		acctgtagatatttgacagcccgg	

**Table 2:** Mouse genetic mutant lines and control genotypes used in each figure

Figure	Abbreviation	KO mice	Source	Genotype of control groups	Comment
Fig. 3	DKO	*Trh* KO mice	Yamada et al. (1997)	*Trh^+/−^*^or^ *^−/−^*; *Oxt^+/−^* ^or^ *^−/−^*	^+/^* means ^+/+^ or *^+/−^*
		*Oxt* KO mice	JAX (#2713)		
Fig. 4	QKO	*Avpr 1a* KO mice	JAX (#5776)	All other genotypes besides *Avpr1a^−/−^*; *Avpr1b^−/−^*; *Trh^−/−^*; *Oxt^−/−^* (Fig. 4*C*)	
		*Avpr 1b* KO mice	JAX (#6160)	*Avpr1a^+/−^* ^or^ *^−/−^*; *Avpr1b^+/−^*; *Trh^+/−^* ^or^ *^−/−^*; *Oxt^+/−^* ^or^ *^−/−^* (Fig. 4*D*)	
		*Trh* KO mice	Yamada et al. (1997)	*Avpr1a^+/−^*; *Avpr1b^+/−^* ^or^ *^−/−^*; *Trh^+/−^* ^or^ *^−/−^*; *Oxt^+/−^*^or^ *^−/−^* (Fig. 4*E*)	
		*Oxt* KO mice	JAX (#2713)	*Avpr1a^+/−^* ^or^ *^−/−^*; *Avpr1b^+/−^* ^or^ *^−/−^*; *Trh^+/−^*; *Oxt^+/−^* ^or^ *^−/−^* (Fig. 4*F*)	
				*Avpr1a^+/−^* ^or^ *^−/−^*; *Avpr1b^+/−^* ^or^ *^−/−^*; *Trh^+/−^* ^or^ *^−/−^*; *Oxt^+/−^* (Fig. 4*G*)	
Fig. 5	TKO	*Avpr 1a* KO mice	JAX (#5776)	All other genotypes besides *Avpr1a^−/−^*; *Avpr1b^−/−^*; *Oxtr^−/−^* (Fig. 5*C*)	
		*Avpr 1b* KO mice	JAX (#6160)	*Avpr1a^+/−^*; *Avpr1b^+/−^*; *Oxtr^+/−^* (Fig. 5*D*)	
		*Oxtr* KO mice	Takayanagi et al. (2005)	*Avpr1a^+/−^* ^or^ *^−/−^*; *Avpr1b^+/−^*; *Oxtr^+/−^* ^or^ *^−/−^* (Fig. 5*E*)	
				*Avpr1a^+/−^*; *Avpr1b^+/−^* ^or^ *^−/−^*; *Oxtr^+/−^* ^or^ *^−/−^* (Fig. 5*F*)	
				*Avpr1a^+/−^* ^or^ *^−/−^*; *Avpr1b^+/−^* ^or^ *^−/−^*; *Oxtr^+/−^* (Fig. 5*G*)	
Fig. 6	TKO	*Avpr 1a* KO mice	JAX (#5776)	*Avpr1a^+/−^*; *Avpr1b^+/−^*; *Oxtr^+/−^*	
		*Avpr 1b* KO mice	JAX (#6160)		
		*Oxtr* KO mice	Takayanagi et al. (2005)		
Fig. 7	TKO	*Avpr 1a* KO mice	JAX (#5776)	All other genotypes besides *Avpr1a^−/−^*; *Avpr1b^−/−^*; *Oxtr^−/−^* (Fig. 7*B*)	
		*Avpr 1b* KO mice	JAX (#6160)	*Avpr1a^+/−^*; *Avpr1b^+/−^*; *Oxtr^+/−^* (Fig. 7*C*)	
		*Oxtr* KO mice	Takayanagi et al. (2005)	*Avpr1a^+/−^* ^or^ *^−/−^*; *Avpr1b^+/−^*; *Oxtr^+/−^* ^or^ *^−/−^* (Fig. 7*D*)	
				*Avpr1a^+/−^*; *Avpr1b^+/−^* ^or^ *^−/−^*; *Oxtr^+/−^* ^or^ *^−/−^* (Fig. 7*E*)	
				*Avpr1a^+/−^* ^or^ *^−/−^*; *Avpr1b^+/−^* ^or^ *^−/−^*; *Oxtr^+/−^* (Fig. 7*F*)	
Fig. 8	HIR	*Avpr1a* and *1b* DKO mice	Kumamoto University (#559)	*Avpr1a^+/−^*; *Avpr1b^+/−^*; *Oxtr^+/−^*	JAX (#5776) *Avpr1a* KO line was not a null mutation, Kumamoto (#599) *Avpr1a* KO line has been confirmed for the phenotype in cardiovascular system	
		*Oxtr* KO mice	Takayanagi et al. (2005)			
Fig. 9	TKO	*Avpr 1a* KO mice	JAX (#5776)	*Avpr1a^+/−^* ^or^ *^−/−^*; *Avpr1b^+/−^*; *Oxtr^+/−^* ^or^ *^−/−^* (Fig. 9*B*)	
		*Avpr 1b* KO mice	JAX (#6160)	*Avpr1a^+/−^*; *Avpr1b^+/−^* ^or^ *^−/−^*; *Oxtr^+/−^* ^or^ *^−/−^* (Fig. 9*C*)	
		*Oxtr* KO mice	Takayanagi et al. (2005)	*Avpr1a^+/−^* ^or^ *^−/−^*; *Avpr1b^+/−^* ^or^ *^−/−^*; *Oxtr^+/−^* (Fig. 9*D*)	
Fig. 10	TKO	*Avpr 1a* KO mice	JAX (#5776)	*Avpr1a^+/−^*; *Avpr1b^+/−^*; *Oxtr^+/−^* (large panels)	
		*Avpr 1b* KO mice	JAX (#6160)	*Avpr1a^+/−^* ^or^ *^−/−^*; *Avpr1b^+/−^* ^or^ *^−/−^*; *Oxtr^+/−^* (small left panels)	
		*Oxtr* KO mice	Takayanagi et al. (2005)	*Avpr1a^+/−^*; *Avpr1b^+/−^^or^^−/−^*; *Oxtr^+/−^* ^or^ *^−/−^* (small middle panels)	
				*Avpr1a^+/−^* ^or^ *^−/−^*; *Avpr1b^+/−^*; *Oxtr^+/−^* ^or^ *^−/−^* (small right panels)	

### Assessment of pup-directed behaviors in standard condition

For pup-exposure assays, 2 d before the first behavioral testing, mice were housed individually in clean cages with purified paper bedding (Alpha-Dri, Shepherd Specialty Papers) and a piece of cotton square (Nestlets, Ancare) as nest material.

Pup-directed behaviors of mice were examined as described (Kuroda et al., 2011). Briefly, either three of their own pups or three unfamiliar pups 1–5 d old (donor pups) were gently introduced to the corner of the home cage of the subject mice avoiding the nest. Behavioral responses toward pups were observed as follows:

Latency to the first sniffing: the latency to when the subject sniffed a pup for the first time in a trial.

Latency to the first pup retrieval: the latency to when the subject retrieved the first pup to the nest.

Latency to the last pup retrieval: the latency to when the subject retrieved the last pup to the nest. If the subject retrieved all three pups to the nest, the retrieval of the third pup was considered as the last pup retrieval. If the subject retrieved two pups to where another pup was located and made a new nest there, the retrieval of the second pup was considered as the last pup retrieval. If the subject did not retrieve any pup, or retrieved only one or two pups and failed to retrieve the rest, the last pup retrieval time was regarded as a missing value.

Total time in nest for three pups: the sum of the duration of each pup being in the nest. If the subject retrieved two pups to where another pup was located and made a new nest there, the pup that was already in the new nest site was regarded as being in the nest since when the first pup was retrieved to the new nest site.

Pup grouping: collecting all three pups in the nest so that the pups contact each other.

Full parental behavior: when the subject exhibited all of the following behaviors within a trial, it was regarded as “full parental behavior”: sniffing, retrieving and grouping all the pups, and crouching over them in the nest for >1 min.

Also, in the QKO, TKO, and HIR analyses, the subjects’ behaviors were coded at 15 s intervals for pup sniffing, pup licking, nest building, retrieval, crouching over pups, and nonpup directed behaviors (such as feeding, resting, running in the cage). In addition, pups’ audible distress calls induced by the subjects’ contacts were noted. If the subject mice started to attack and bite the pups, all pups were immediately removed from the cage, and the subjects were deemed as “infanticidal.” The attacked pups were immediately euthanized. For the QKO and TKO mothers (Figs. 4, 5), the observation period was separated into two periods. The mother mice were observed for 15 min just after introduction of pups (first observation of the day); and then after a 15-min resting period with the introduced pups, they were again observed for 15 min (second observation of the day). The duration data from the first 15 min observation periods was used for analyses.

**Figure 3.**
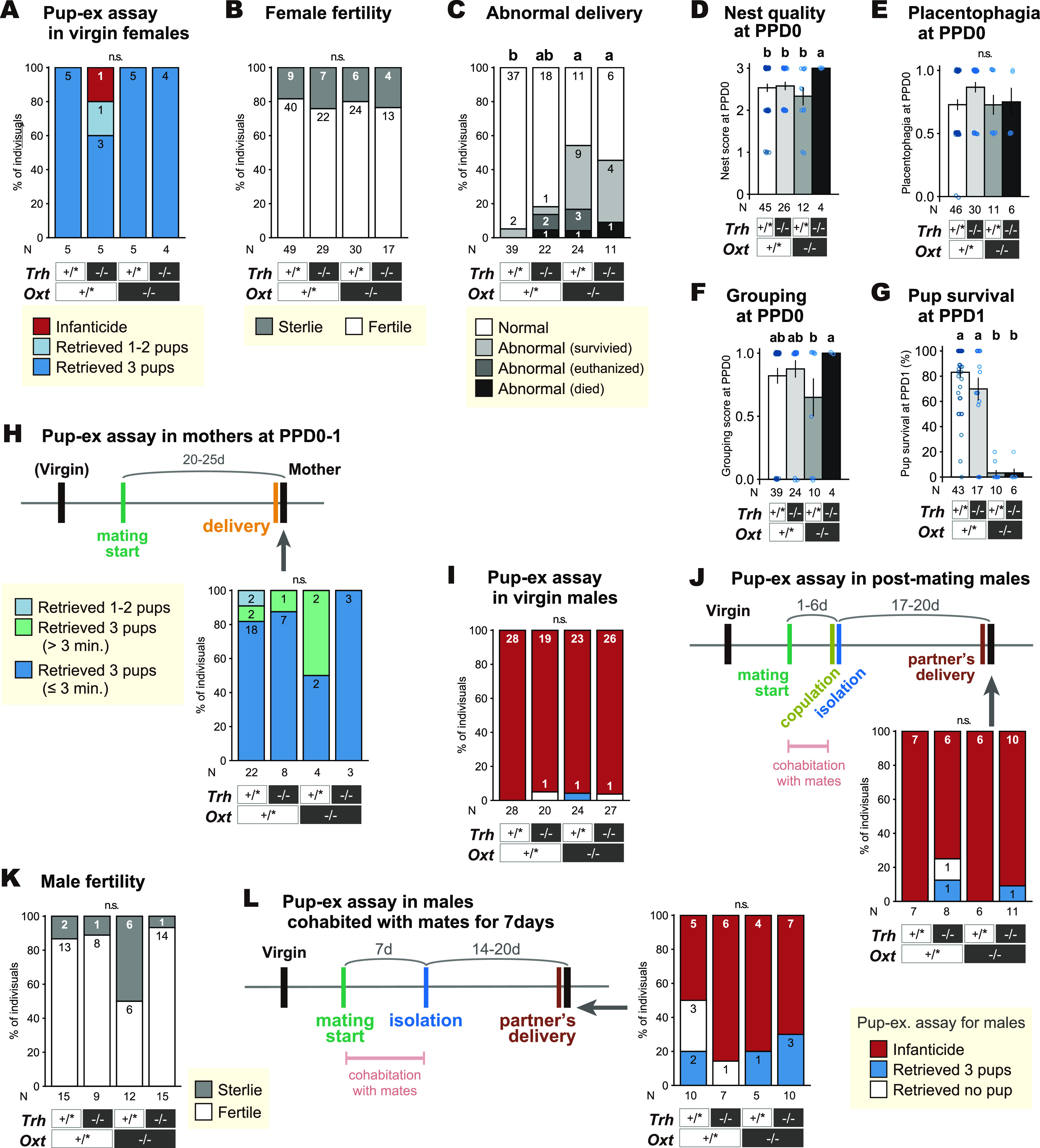
Parental behavior, fertility, and abnormal delivery of the *Trh-Oxt* double knock-out (DKO) mice. ***A***, Responses to pup exposure in virgin females. ***B***, Fertility ratio of the females after mating. ***C***, Proportion of abnormal deliveries (prolonged for >24 h, and/or maternal distress with remaining pups in uterus, which sometimes caused maternal death in labor). “Abnormal (died)” means maternal death by PPD3, and “abnormal (euthanized)” means maternal health deterioration necessitating euthanasia by PPD3. ***D***, The scores for nest quality, indicated by the use of nest material and nest shape (see Materials and Methods) at PPD 0. ***E***, The placentophagia score (1, all the live pups were cleaned for amniotic membrane, umbilical cord, and placenta; 0.5, partially; 0, none of the pups were cleaned) at PPD 0. ***F***, The spontaneous pup grouping score (1, all the live pups were grouped in the nest; 0.5, the live pups were in the nest except one; 0, all the live pups were outside of the nest.) at PPD 0. ***G***, The pup survival rate (number of live pups at PPD 1 divided by the number of live pups at PPD 0 morning) of each mother. ***H***, Schematic of the experimental timeline and responses to pup exposure in mothers. Only four of them (*Oxt*^+/+^-*Trh^+/−^*: 1, *Oxt*^−/−^-*Trh^+/−^*: 2, *Oxt*^−/−^*Trh*^−/−^: 1) had experienced the pup-exposure assay as a virgin, whose results are included in ***A***. ***I***, Responses to pup exposure in virgin males. ***J***, Responses to pup exposure in males, which were separated from their female mates immediately after copulation. Copulation was assessed by vaginal plugs in females. Only males whose mates were later confirmed as pregnant are shown. ***K***, Fertility ratios of the males after mating (7 d of cohabitation with females). ***L***, Responses to pup exposure in males, which were separated from their female mates after 7 d of cohabitation. For ***D–H***, data from only mothers who had normal delivery are shown. For ***J–L***, data from only males who exhibited infanticide as a virgin are shown. (+/*) means wild-type (+/+) or heterozygous KO (*+/−*), and (−/−) means homozygous KO for the designated genetic locus. A black vertical bar in schematic of the experimental timelines indicates the day a pup-exposure assay was performed. Error bars represent the mean ± SEM. Different letters denote significant differences (multiple comparisons by Fisher’s exact test for categorical data, and Welch’s *t* test for continuous data), *p *<* *0.05. Numbers of animals used are described in the figure.

For the *Oxt-Trh* DKO mice, four of the females used for the analyses of pup-directed behaviors were tested twice as a virgin, but only the results from the first trials were analyzed (Fig. 3*A*). These four females were not used for the further analyses. Other virgin females were tested only once as a virgin. Of the DKO females used for the analyses of postpartum pup-directed behaviors, only four were experienced the pup-exposure test as a virgin (whose results were included in Fig. 3*A*). The postpartum tests were performed either on the day of delivery or 1 d later. All the *Oxt-Trh* DKO male mice were tested once as virgins (Fig. 3*I*). Only males who exhibited infanticidal behavior as virgins were used for further analysis. Males were then cohabited with females. Some of these males were separated from their mates immediately after copulation (assessed by vaginal plugs), and tested on the day the paired females delivered (Fig. 3*J*). The other males were separated from their female mates after 7 d of cohabitation, and tested on the day the paired females delivered (Fig. 3*L*).

**Figure 4.**
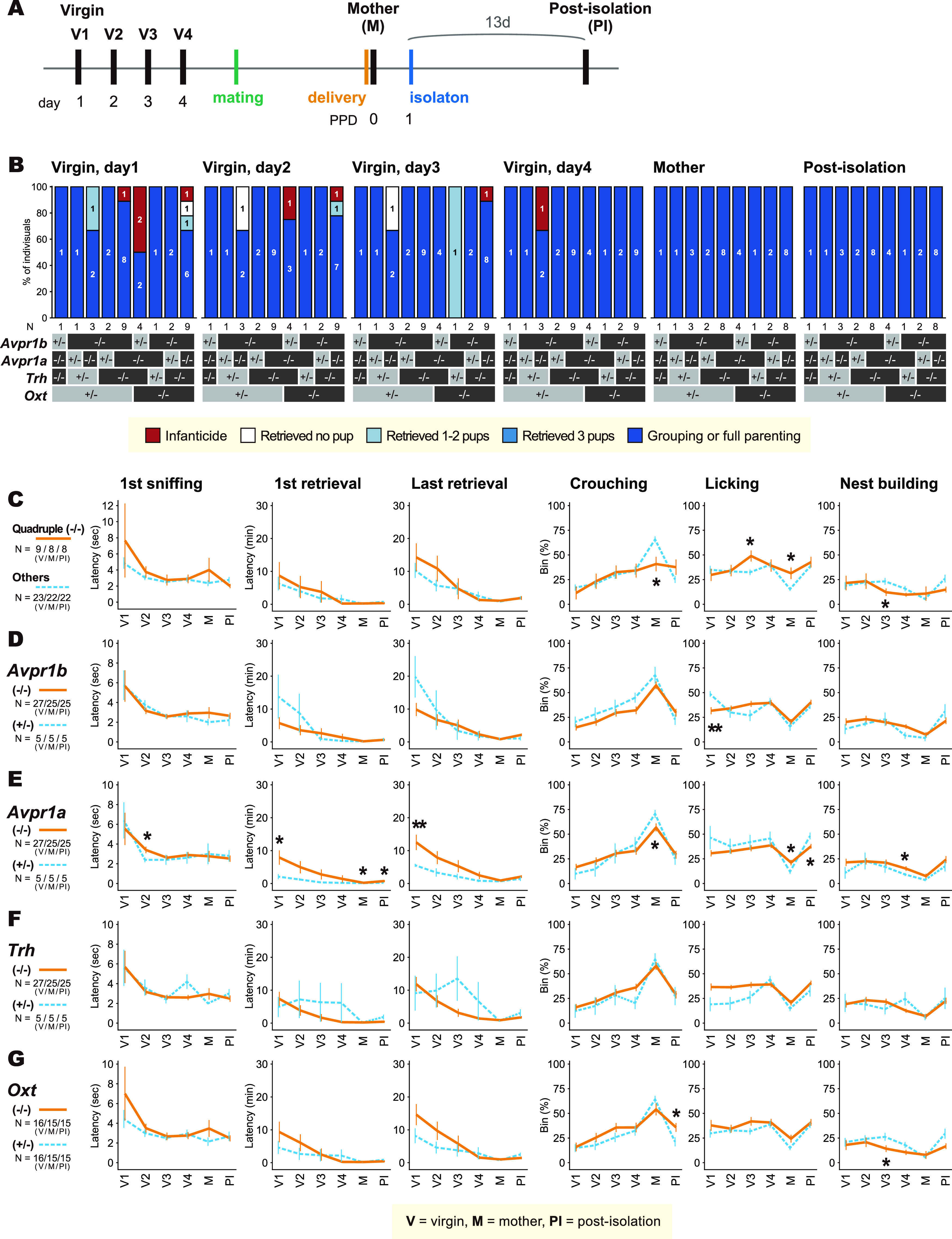
Pup-directed behaviors of the virgin and postpartum *Oxt*-*Trh*-*Avpr1a*-*Avpr1b* quadruple knock-out (QKO) female mice in the standard pup-exposure assay. ***A***, Schematic of the experimental timeline. “Posti-solation (PI)”: the mothers tested 13 d after the isolation from pups at PPD1. A black vertical bar indicates the day a pup-exposure assay was performed. ***B***, Gross pup-directed behaviors in each genotype, with the number of mice for each behavioral category in each genotype. ***C–G***, Details of pup-directed behaviors compared between quadruple (−/−) versus all other genotypes (others; ***C***); heterozygous (*+/−*) versus homozygous KO (−/−) for *Avpr1b* (***D***), *Avpr1a* (***E***), *Trh* (***F***), and *Oxt* (***G***), regardless of other genetic loci. The left three variables on the abscissae are latencies, the right three variables are proportions of the time bins in which the indicated behaviors were performed. Error bars represent the mean ± SEM. Fisher’s exact test for categorical data; Welch’s *t* test for continuous data; **p <* *0.01, *p <* *0.05. Numbers of animals used are described in the figure.

**Figure 5.**
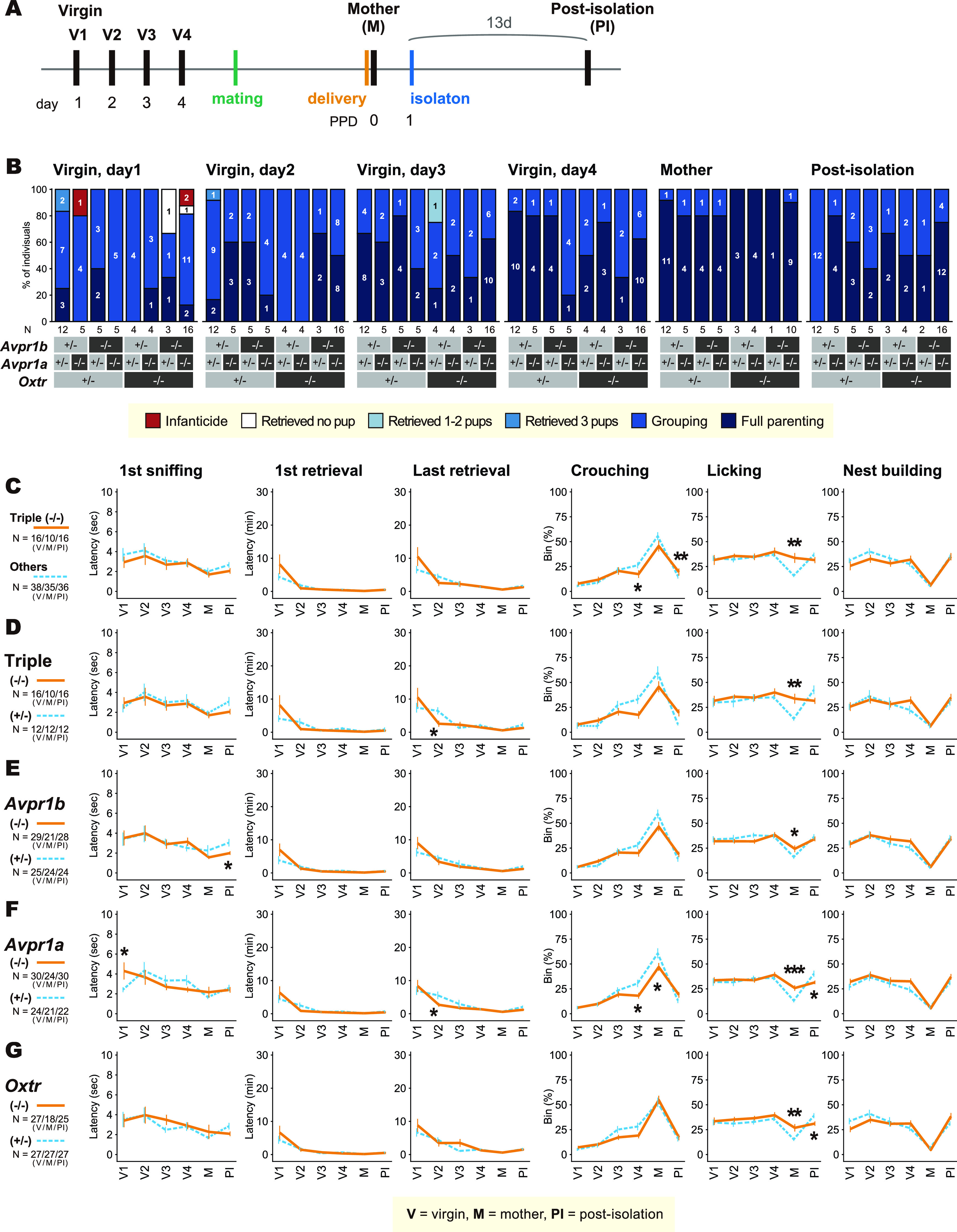
Pup-directed behaviors of the virgin and postpartum *Oxtr-Avpr1a-Avpr1b* triple knock-out (TKO) female mice. ***A***, Schematic of the experimental timeline. “Post-isolation (PI)”: the mothers tested 13 d after the isolation from pups at PPD1. A black vertical bar indicates the day a pup-exposure assay was performed. ***B***, Gross pup-directed behaviors in each genotype, with the number of mice for each behavioral category in each genotype. ***C–G***, Details of pup-directed behaviors compared between triple (−/−) versus all other genotypes (others; ***C***); triple (−/−) versus *Oxtr^+/−^*; *Avpr1a^+/−^*; *Avpr1b^+/−^* [triple (*+/−*); ***D***], *Avpr1b* (***E***), *Avpr1a* (***F***), and *Oxtr* (***G***), regardless of other genetic loci. The left three variables on the abscissae are latencies, the right three variables are proportions of the time bins in which the indicated behaviors were performed. Error bars represent the mean ± SEM. Fisher’s exact test for categorical data; Welch’s *t* test for continuous data; ***p <* *0.001, **p <* *0.01, *p <* *0.05. Numbers of animals used are described in the figure.

For the QKO and TKO female mice (Figs. 4, 5), the responses to pups were first examined once per day for four straight days. Each female was subsequently paired with a sexually-experienced male C57BL/6J mouse to allow mating and delivery. The male mouse was changed to another in the case that the female did not become pregnant by three weeks cohabitation. On the day of delivery, the newborn pups were removed, and their location in the cage, general health, milk band, and remaining fetal tissues were briefly examined according to the previous study (Kuroda et al., 2011). Then using their own three pups, the subject mothers were observed for their behavior as described above. In the case that the newborn pups were not cleaned properly, unhealthy, or dead, donor pups were used instead. Use of donor pups does not significantly affect pup exposure assay (Kuroda et al., 2011). If the mother showed any apparent abnormality of their movement caused by their parturition (observed in *Oxt* and *Oxtr* KO mice), we did not conduct the behavioral test on the day of delivery. On the next morning (PPD1), the resident pups in the home cage were gently removed and their conditions were checked again as they had been the day before (day of delivery). Then, the female mice were totally isolated from the pups. After the isolation, they were subjected to behavioral testing once again 13 d later.

For the TKO male mice and their littermates (Fig. 7), the responses to pups were examined once before mating. Each male was subsequently paired with two parous C57BL/6J mice to allow mating. They were checked for vaginal plugs daily, and the cohabitation with females continued for 17 d after confirmation of the plugs to assure pregnancy as indicated by enlargement of abdomen. In the case that a female become pregnant without a plug, both females were removed from the mating cage. If there were no sign of pregnancy after a two-month cohabitation, the males were not used for the subsequent tests. One to 2 d after one of the paired females delivered, the male behavior was examined using their biological offspring once per day for two straight days, and once again 12–14 d later.

For the HIR female mice (Fig. 8*A*,*B*), different individuals were used for virgin female and mother mice assays. For virgin females, the responses to pups were first examined once per day for three straight days. For PPD0 mothers, they were subjected to behavioral testing on the day of delivery as described above, but with three donor pups instead of their own. After the testing, mother mice were totally isolated from the pups. Then they were subjected to behavioral testing for two straight days. For the HIR male mice (Fig. 8*C*,*D*), different individuals were used for virgin male and father mice assays. For virgin males, the responses to pups were first examined once per day for three straight days. For father mice, they were cohabitated with female and own pups for 2 d after the delivery. Then, the father mice were totally isolated from the pups. They were subjected to behavioral testing once per day for three straight days, using three donor pups.

### Assessment of pup-directed behaviors after restraint stress

Pup-directed behaviors immediately after acute restraint stress were first examined once per day for four straight days using virgin female TKO mice (Fig. 9). Restraint stress was given for 30 min using well-ventilated 50 ml conical tubes. The mice were released into their own home cages after three unfamiliar pups of 1–5 d old were gently introduced to the corner of the cage of the subject mice avoiding the nest. Behavioral responses toward pups were observed in the same way as in the TKO virgin female assays under standard conditions as described in the previous section.

### Assessment of stress sensitivity

**Figure 6.**
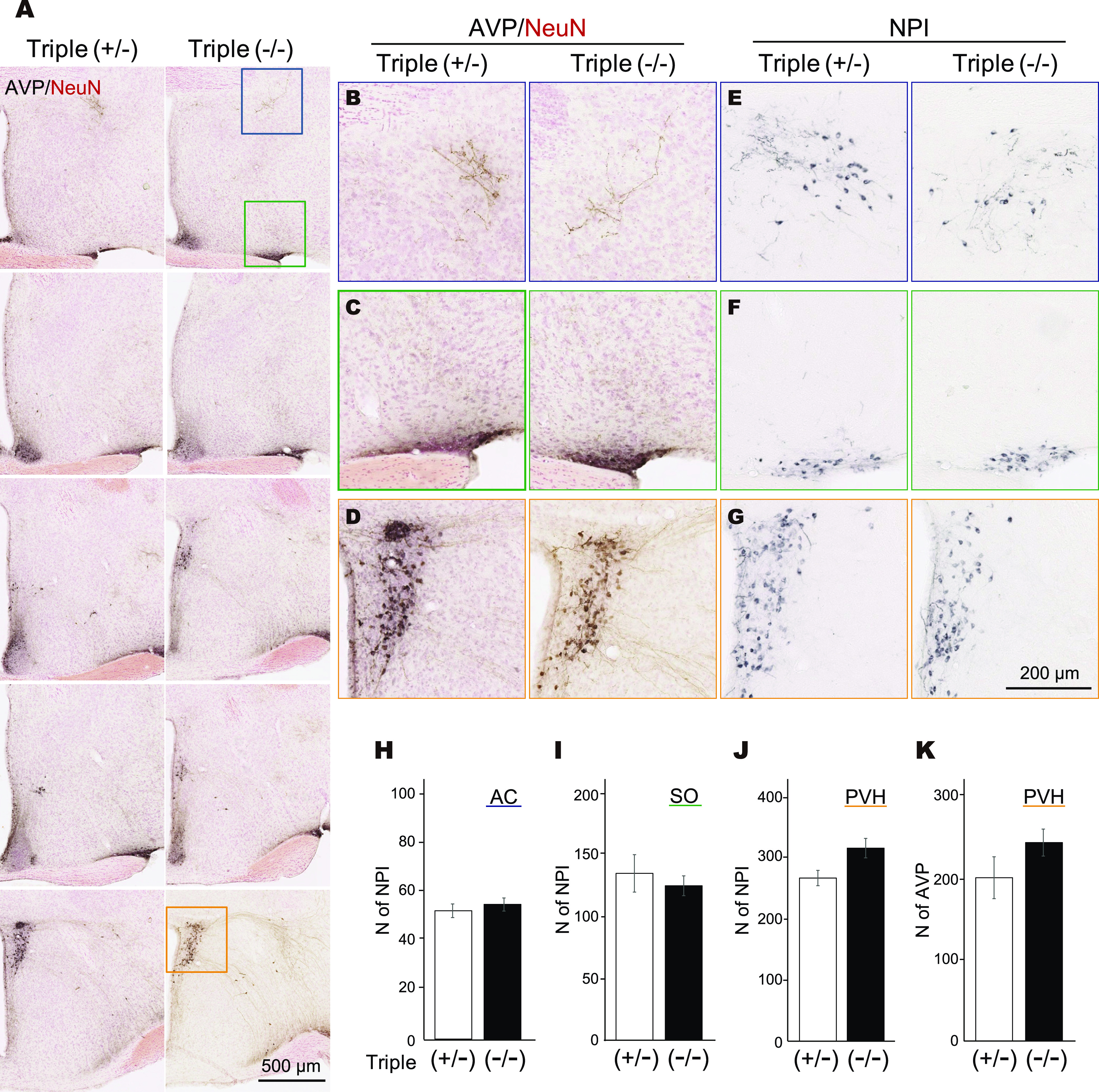
Oxt (NPI) and AVP expressions in the MPOA were not affected by *Oxtr* or *Avpr* genetic targeting. Distribution of NPI and AVP (black)-ir cells in the MPOA of *Avpr1a-Oxtr-Avpr1b* triple mutant (TKO) female mice; triple (+/−) mean *Avpr1a^+/−^*^;^ *Oxtr^+/−^*; *Avpr1b^+/−^*, triple (−/−) mean *Avpr1a*^−/−^; *Oxtr*^−/−^; *Avpr1b*^−/−^ TKO virgin female mice. ***A***, The sections were stained by IHC. AC (***B***, ***E***), SO (***C***, ***F***), and PVH (***D***, ***G*** was identified by counterstain using NeuN). All panels are arranged in anterior–posterior order. ***B–G***, High-magnification images of same-colored squares. ***H–K***, The numbers of NPI-ir neurons in the AC (***H***), SO (***I***), and PVH (***J***) and of Avp-ir neurons in the PVH (***K***) of triple (*+/−*; *N* = 4) and (−/−; *N* = 4) virgin females. Error bars represent the mean ± SEM.

**Figure 7.**
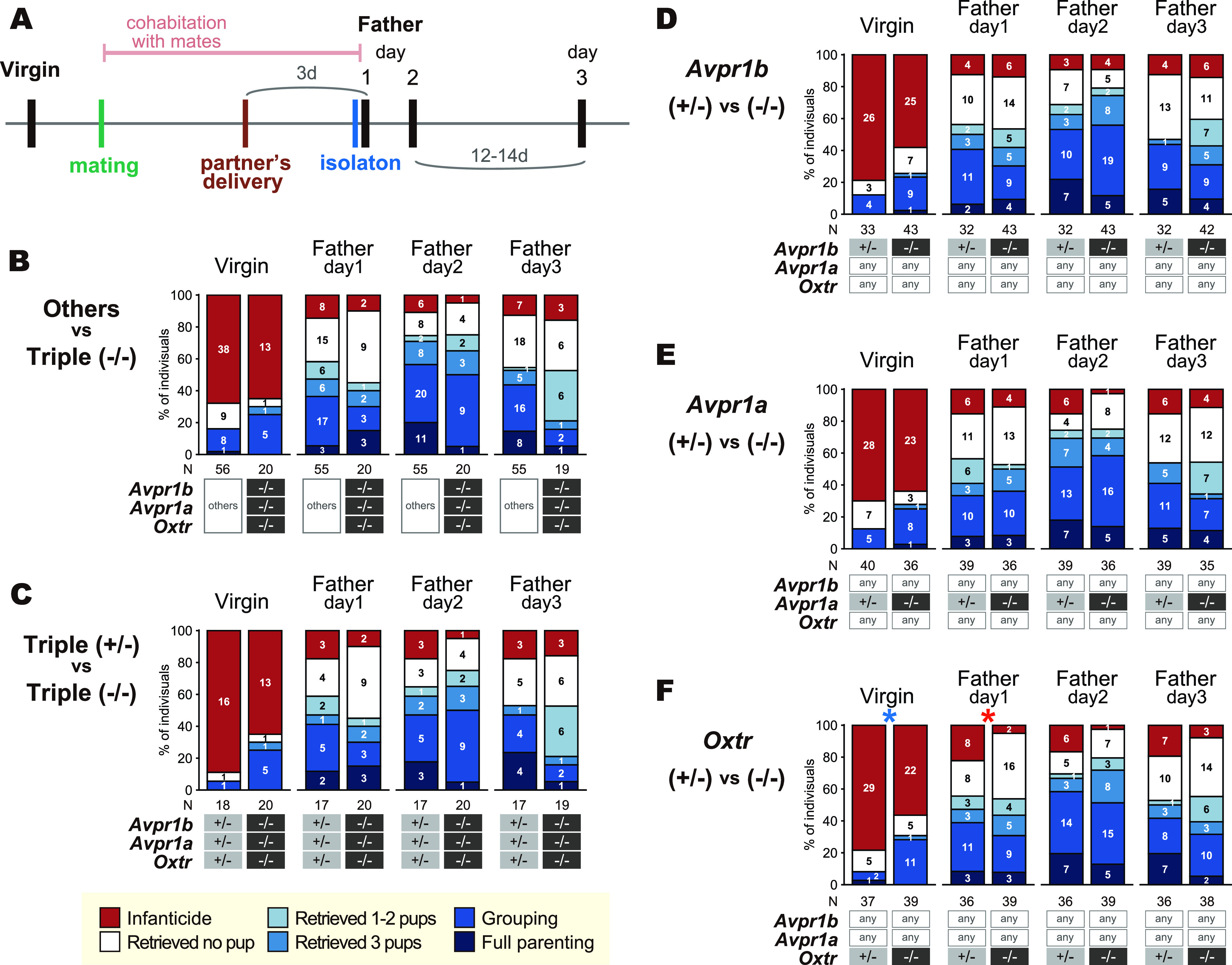
Responses to pups in the standard pup-exposure assay with the virgin and copulated *Oxtr-Avpr1a-Avpr1b* triple knock-out (TKO) male mice. ***A***, Schematics of the experimental timeline. A black vertical bar indicates the day a pup-exposure assay was performed. ***B–F***, Gross pup-directed behaviors in each genotype, with the number of mice for each behavioral category in each genotype. *Oxtr*^−/−^; *Avpr1a*^−/−^; *Avpr1b*^−/−^ [triple (−/−)] versus all other genotypes (others; ***B***); triple (−/−) versus *Oxtr^+/−^*; *Avpr1a^+/−^*; *Avpr1b^+/−^* [triple (*+/−*); ***C***]; heterozygous (*+/−*) versus homozygous (−/−) KO for each four genetic loci for *Avpr1b* (***D***), *Avpr1a* (***E***), and *Oxtr* (***F***), regardless of other genetic loci. Blue asterisk: Fisher’s exact test between parental (pups retrieved) versus nonparental (infanticide or no pup retrieved); red asterisk: Fisher’s exact test between infanticidal versus noninfanticidal; *p <* *0.05.

**Figure 8.**
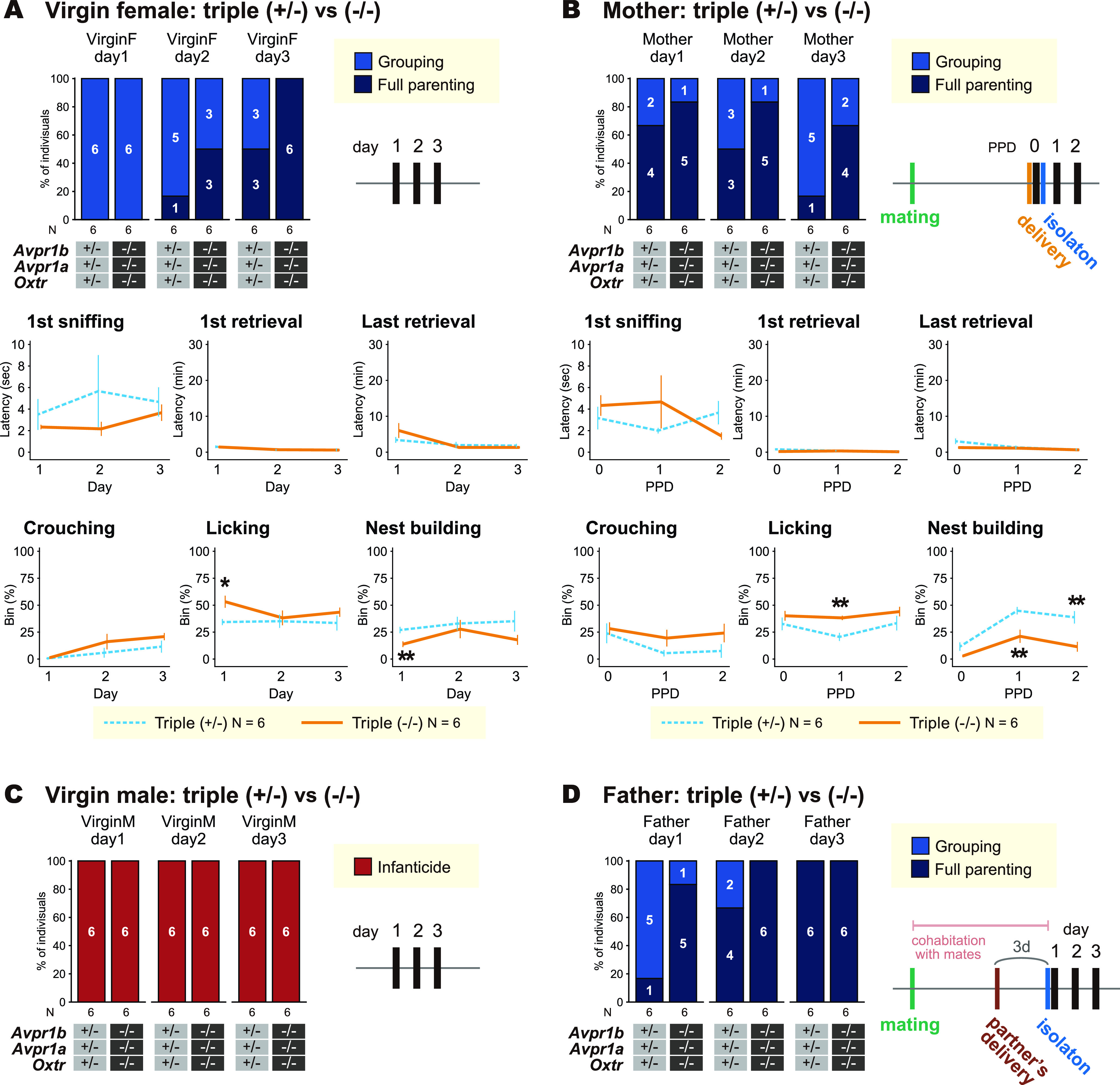
Confirmation of the results with HIR strain of *Oxtr-Avpr1a-Avpr1b* triple knock-out (TKO) mice. ***A***, ***B***, Responses to pups, schematics of the experimental timeline, latencies, and proportion of time of pup-directed behaviors in the standard pup-exposure assay in virgin (***A***) and postpartum (***B***) females. The variables in the second rows are latencies, the variables in the third rows are proportion of time bins performed the indicated behavior. ***C***, ***D***, Responses to pups and schematic of the experimental timeline of pup-directed behaviors in the standard pup-exposure assay in virgin males (***C***) and father mice (***D***). A black vertical bar in schematic of the experimental timelines indicates the day a pup-exposure assay was performed. Error bars represent the mean ± SEM. Fisher’s exact test for categorical data; Welch’s *t* test for continuous data; **p <* *0.01, *p <* *0.05. Numbers of animals used are described in the figure.

Phenotypic behavioral differences between *Oxt^+/−^* and *^−/−^*, *Avpr1a^+/−^* and ^−/−^, and *Avpr1b^+/−^* and *^−/−^* mice were assessed with a specific behavioral and physiological test battery consisting of open-field (OF) and elevated plus maze (EPM) test using virgin male and female TKO mice for Figure 10. The cohort of mice were different from those in Figures 5-8. The OF and EPM tests were conducted basically as previously described (Kuroda et al., 2008) with minor modifications as described below. The effects of restraint stress on plasma corticosterone (CORT) levels were also examined.

#### Open field (OF) test

The subjects were individually housed 2 d before the experiment, and were given white noise on the day before the experiment. An OF monitoring system equipped with four monitoring channels was used (O’Hara & Co, Ltd.). Mice were placed in the center of the OF (50 × 50 cm, 40-cm-high gray acrylic walls, bright-light condition of 70 lux) and allowed to explore for 15 min. The distance traveled and precent of time at the center area of the field (size is 36% of the field) were measured using an automatic monitoring system Time OFCR4 (O’Hara & Co, Ltd.).

#### Elevated plus maze (EPM) test 

A week after the OF test, the mice were tested in the EPM for 15 min. The maze was set at a height of 50 cm above the floor and consisted of four arms (25 × 5 cm), and a platform made of gray acrylic: two opposite arms were open, and the other two arms were enclosed by 15-cm-high transparent walls (room was illuminated at 70 lux). A mouse was placed in the center platform, positioned to face one of the open arms, and allowed to explore the maze for 15 min. The time spent in the different arms and the numbers of arm entries were automatically analyzed using Time EP2 Two Maze System (O’Hara & Co, Ltd.).

#### Restraint stress and plasma CORT

A week after the EPM assessments, half the subject mice were given acute restraint stress for 30 min using well-ventilated 50-ml conical tubes. The rest of mice were left without stress for 30 min. Then the mice were decapitated and the trunk blood immediately collected was assayed for CORT by ELISA (Corticosterone, ELISA kit, AssayMax; ASSAYPRO). Blood collection was performed in the morning, between 10 A.M. and 12 P.M.

### Preparation of brain sections

For histologic analyses in Figure 6, TKO mice used for the Figure 5 experiments were deeply anesthetized with sodium pentobarbital (50 mg/kg, i.p.) and then perfused transcardially with 4% (w/v) paraformaldehyde (PFA) in PBS (pH 7.4). The brains were removed, immersed in the same fixative at 4°C overnight, followed by cryoprotection in the series of 20% and 30% (w/v) sucrose in PBS for 2 d, embedded in O.C.T. compound (Sakura Finetek Japan), and stored at −80°C until cryosectioning. Brains were cryosectioned coronally at a thickness of 40 μm and evaluated using the mouse brain atlas (Franklin and Paxinos, 2007). Every third section from the serial sections was processed for immunohistochemistry (IHC).

### Immunohistochemistry (IHC)

IHC on free-floating sections was performed essentially as described (Tsuneoka et al., 2013). Single and double labeling was performed for immunohistochemical detection of Neurophysin I (NPI), Avp, c-Fos, and NeuN. To label Oxt neurons, anti-NPI was used, as Oxt and NPI are cleaved from the same precursor, preprooxyphysin. The anti-NPI antibody is more sensitive than an anti-Oxt antibody. The sections were washed with PBS containing 0.2% Triton X-100 (PBST), incubated with 0.3% H_2_O_2_ in methanol for 5 min, washed with PBST, blocked with 0.8% Block Ace (Dainihon-Seiyaku) in PBST, and incubated at 4°C overnight with goat primary antibody against NPI (1:6000, sc-7810, Santa Cruz Biotechnology). The following morning, the sections were washed and incubated with biotin-conjugated horse anti-goat secondary antibody (1:2000, BA-9500, Vector Laboratories) for 2 h and then in ABC peroxidase reagent (Vectastain ABC Elite kit; Vector Laboratories) for 1 h according to the manufacturer’s instructions. The labeling was visualized by incubation in 3,3′-diaminobenzidine (DAB) solution with nickel intensification (DAB peroxidase substrate kit, Vector Laboratories) for 5 min. For c-Fos-NPI and Avp-NeuN double staining, the first staining was processed similarly except the staining procedure used rabbit primary antibody against c-Fos (1:5000, sc-52, RRID:AB_2106783, Santa Cruz Biotechnology) or Avp (1:5000, AB1565, Millipore), and biotin-conjugated horse anti-rabbit secondary antibody (1:2000, BA-1100, Vector Laboratories). The sections underwent the second staining, which used anti-NPI antibody, mouse anti-NeuN antibody (1:6000, MAB377, Millipore Corporation). For anti-NeuN double staining, horse anti-mouse secondary antibody (1:2000, BA-2000, Vector Laboratories), and the ABC alkaline phosphatase reagent (Vectastain ABC-AP kit, Vector Laboratories) were used. The brown signals were developed by 5 min of immersion in DAB solution without nickel, and pink color was developed by 5 min of immersion in Vector Red substrate (Vector red alkaline phosphatase substrate kit, Vector Laboratories). Subsequently, they were washed with PBS and then mounted on gelatin-coated slides using mounting medium (Vectashield; Vector Laboratories).

### Histologic analysis

For labelling of NPI-immunoreactive (ir) and c-Fos-ir cells, three rostral-to-caudal sections where the NPI and c-Fos expression were prominent were examined. Double-labeled sections of c-Fos and NPI were viewed under a brightfield microscope (Leica DM6000B; Leica Microsystems). The labeling protocol was described (Tsuneoka et al., 2013).

For analysis of NPI-ir and Avp-ir cells of TKO female mice (Fig. 6), brightfield photomicrographs were obtained using a digital slide scanner (NanoZoomer Digital Pathology; Hamamatsu Photonics) with a 20–40× objective. The contrast and brightness of the all photographs were adjusted only linearly and uniformly for all the micrographs used in one experiment, using software (ImageJ; Rasband, 1997–2018). Captured images were manually labeled by ImageJ. Paraventricular hypothalamic nucleus (PVH) and supraoptic nucleus (SO) were identified by counterstaining using NeuN-ir. Anatomical nomenclature and classification were used according for reference (Watson et al., 2011).

### Statistical analysis

**Figure 9.**
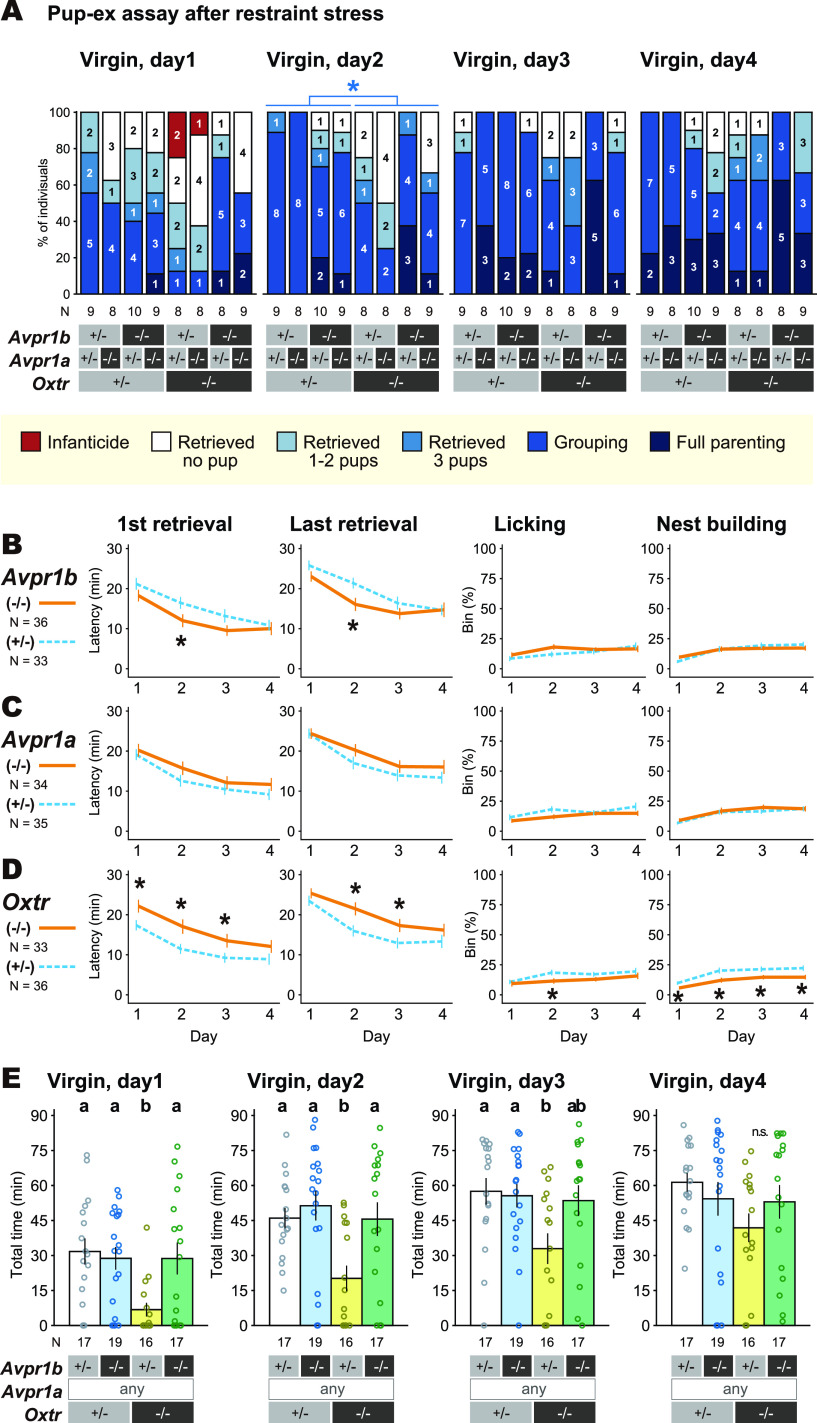
Effect of restraint stress on the pup-directed behaviors of the virgin and postpartum *Oxtr-Avpr1a-Avpr1b* triple knock-out (TKO) female mice. ***A***, Gross pup-directed behaviors in each genotype, with the number of mice for each behavioral category in each genotype. Blue asterisk: Fisher’s exact test between parental (pups retrieved) versus non-parental (infanticide or no pup retrieved), *p <* *0.05. ***B–D***, Details of pup-directed behaviors compared between heterozygous (*+/−*) versus homozygous KO (−/−) for *Avpr1b* (***B***), *Avpr1a* (***C***), and for *Oxtr* (***D***), disregarding other genetic loci. The left two variables are latencies, the right two variables are proportion of time bins performed the indicated behavior. Welch’s *t* test for continuous data; *p <* *0.05. ***E***, Total amount of pup retrieval behavior, measured by the total time the three pups spent in the nest. Different letters denote significant differences (multiple comparisons by Welch’s *t* test, *p *<* *0.05). See also the Extended Data Figure 9-1 for the results of Kruskal–Wallis rank-sum tests with *post hoc* multiple comparisons by Nemenyi test. Error bars represent the mean ± SEM.

All statistical analyses were conducted using R v. 3.6.3 (R Development Core Team, 2018). Welch’s *t* test and Fisher’s exact test were used to compare continuous and categorical data, respectively. Multiple comparisons were conducted with P-values adjusted by Holm’s method. Error bars are mean ± SEM unless otherwise specified. Datapoints of “censored,” incomplete retrieval observations were replaced by the maximum observation time (30 min for virgin females, virgin males, and fathers in Figs. 4, 5, 7, 9, and mothers in Fig. 8; 15 min for mothers in Figs. 4, 5), e.g., if a subject mouse retrieves only one pup within the session, the latencies for the second and third retrieval are regarded as 30 min, rather than as missing datapoints. We also tried survival analyses, but comparing the effects of three genetic loci became complicated in the survival analysis. Please see also the Extended Data Fig. 9-1 for another strategy, and the main conclusion is the same). The biological replicate number is the same as the number of samples, and the technical replicates are not applicable or taken in this study. All the statistical details and the numbers of animals used can be found either in the figure legends or Results.

**Figure 10.**
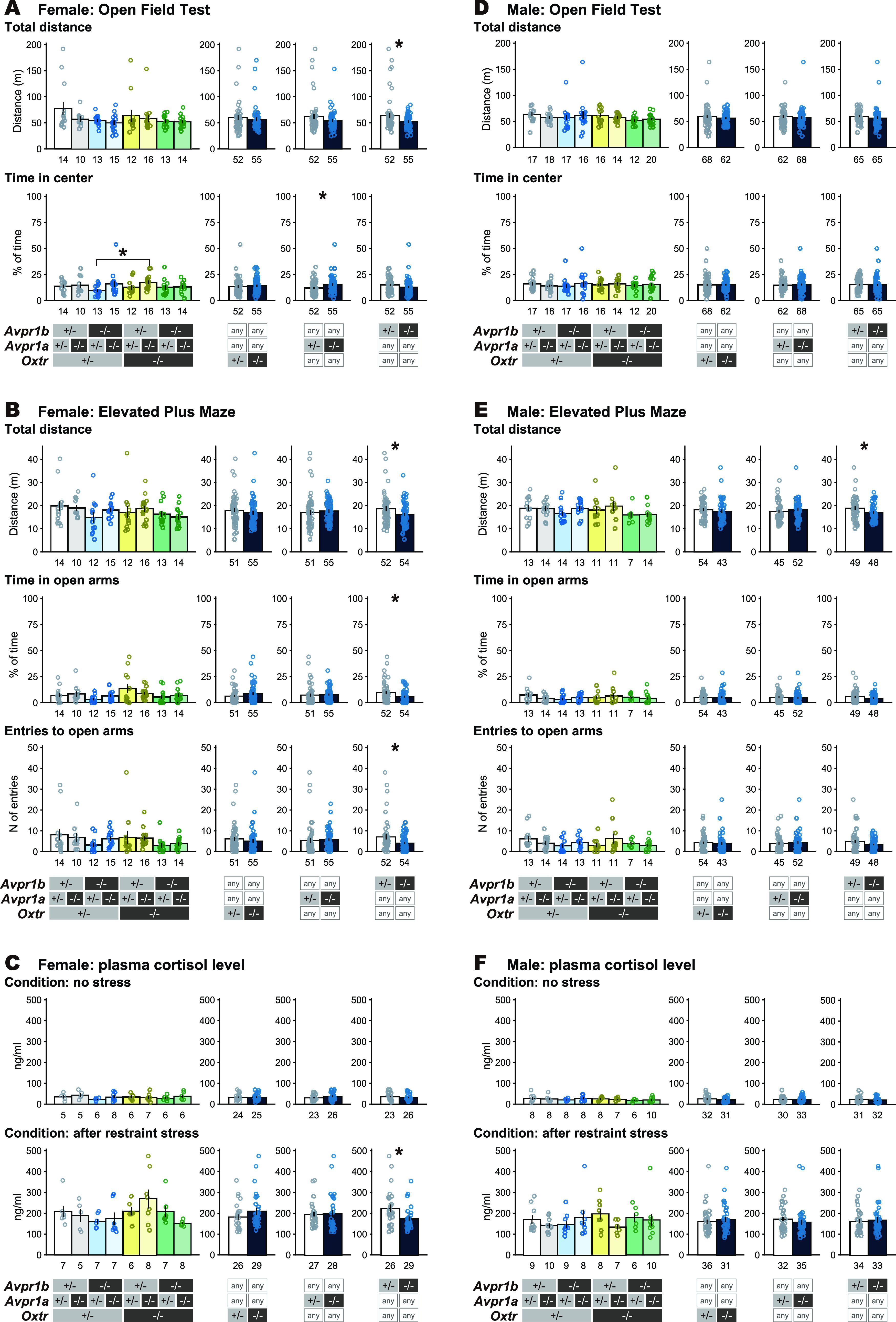
Stress sensitivity of the virgin *Oxtr-Avpr1a-Avpr1b* triple knock-out (TKO) female and male mice. Behavioral characteristics in the open field (OF) test (***A***, ***D***) and elevated plus maze (EPM) (***B***, ***E***), and effect of restraint stress on plasma CORT level (***C***, ***F***). (*+/−*) means heterozygous, (−/−) means homozygous, and * means (*+/−*) or (−/−) KO for the designated genetic locus. Error bars represent the mean ± SEM. Welch’s *t* test, *p <* *0.05. Numbers of animals used are described in the figure.

10.1523/ENEURO.0405-21.2022.f9-1 Extended Data Figure 9-1 Effects of restraint stress on the pup-directed behaviors of the virgin *Oxtr-Avpr1a-Avpr1b* (TKO) triple mutant female mice with nonparametric statistical analysis. Parental behavior, measured by the total time the 3 pups spent in nest, of *Oxtr-Avpr1a-Avpr1b* triple mutant virgin female mice in standard condition (a) and after restraint stress (b). (+/−) means heterozygous, (−/−) means homozygous, and * means (+/−) or (−/−) for the designated genetic locus. *HH means (Avpr1a^*^, *Oxtr*^+/−^, *Avpr1b* ^+/−^; gray with white stripe), *KH means (Avpr1a^*^, *Oxtr^−/−^*, *Avpr1b* ^+/−-^; black with white stripe), *HK means (Avpr1a^*^, *Oxtr*^+/−^, *Avpr1b ^−/−^*; gray), and *KK means (Avpr1a^*^, *Oxtr^−/−^*, *Avpr1b ^−/−^*; black). Results of nonparametric Kruskal–Wallis rank-sum tests are shown above the graphs. # and ## denote significant differences between two groups in *post hoc* multiple comparisons (Nemenyi test; # *p *<* *0.05, ## *p *<* *0.01). Nemenyi tests were performed by the R package *PMCMR* ver. 4.3 (Pohlert, 2014). The box plots were generated by *geom_boxplot* function with default settings in R package *ggplot2* ver. 3.1.0 (Wickham, 2016). The medians are represented by the horizontal bar in the middle of each box. The lower and upper hinges correspond to the first and third quartiles (the 25th and 75th percentiles). The upper whisker extends from the hinge to the largest value no further than 1.5 * IQR from the hinge. The lower whisker extends from the hinge to the smallest value at most 1.5 * IQR of the hinge. Data beyond the end of the whiskers are called “outlying” points and are plotted individually (Wickham, 2016). Download Figure 9-1, EPS file.

## Results

### Neuroanatomical relations of the activated neurons during alloparenting of Oxt neurons and fibers in the preoptic and adjoining areas

Our initial motivation to study the relations between Oxt and parental behavior derived from the remarkable spatial correlation of neuronal activation after pup nurturing with the distribution of oxytocinergic neurons and fibers in the MPOA (Figs. 1, 2). We have already reported the quantification of each MPOA subregions for nurturing-induced activation (Tsuneoka et al., 2013), and here we illustrate anatomic and spatial relations between these neuronal activation with Oxt neurons and fibers. Observing c-Fos expression pattern, we found that parasagittal and horizontal sections consistently depicted the anatomic structures of the PVH (arrowheads), AC (Fig. 1*A*,*B*, box), and the c-Fos neuronal distributions after 2 h (Fig. 1*A*,*B*) or 6 h (Fig. 2) of pup nurturing (arrows) in C57BL/6 virgin female mice. c-Fos-ir neurons were found most densely in the AC, compared with the PVH, SO, and other MPOA subregions. Within the AC, Oxt-ir thick dendrites with a characteristic corkscrew-like morphology and irregular varicosities (Fig. 1*B*, arrows) were numerous (Castel and Morris, 1988). Even outside of the AC, the regions with dense c-Fos-ir neurons were colocalized with Oxt fibers, particularly in the preoptic area but not in the hypothalamus (for example, areas indicated by arrows in the ventral MPOA, Fig. 1, left, second; Fig. 2, left, first and second panels), indicating the spatial correlation between the non-Oxt MPOA neurons involved in nurturing behaviors and the Oxt neurons involved in parturition and lactation. In contrast, there were several spherical tissue masses (Figs. 1*A*, 2, numbered as 1–4) devoid of both c-Fos and Oxt fibers, suggesting that the developmental formation of the parenting-relevant anatomic structure was intermixed with other cell masses in the MPOA. Specifically, the tissue mass 1 roughly corresponds with the ventral part of the septohypothalamic area, mass 2 to the medial preoptic nucleus, mass three overlaps with the striohypothalamic area, and mass four overlaps with the posteroventral MPOA and the ventral part of anterior hypothalamic nucleus. This unique organization might be formed by the embryonic preoptic-strial migration stream (Bayer and Altman, 2004).

In these virgin females, that were not suckled during brief pup nurturing, the induced c-Fos-ir were limited to nonoxytocinergic neurons. There were scarce c-Fos-ir Oxt neurons in the analyzed area that included the AC, PVH, PVPOA, and SO (Figs. 1*A*,*B*, 2) of the pup-exposed virgin females (c-Fos-ir cells/Fos + Oxt double-ir cells = 0.067%, Oxt-ir cells/c-Fos + Oxt double-ir cells = 0.915%), in harmony with previous studies (Tsuneoka et al., 2013; Okabe et al., 2017). Sheehan and colleagues found significant c-Fos induction in PVH by pup exposure (Sheehan et al., 2000), although they included the AC as the anterior magnocellular part of PVH). It should be noted, however, that Fos expression itself does not imply the function of Oxt neurons. For example, while Oxt neurons are shown to be functional in facilitating social contacts among females, only ∼1% of Oxt neurons (30% of parvocellular Oxt neurons, which are ∼3% of total Oxt neurons) in the PVH become c-Fos positive during social contacts (Tang et al., 2020).

### Delayed delivery of *Oxt* KO mice, and grossly-normal pup-directed behaviors of *Trh-Oxt* double mutant mice

The above observations prompted us to directly address the role of Oxt and Avp systems in pup nurturing behaviors in mutant mice. Because normal maternal care was shown previously in three independent genetically targeted mouse lines of *Oxt* (Nishimori et al., 1996; Young et al., 1996; Gross et al., 1998), we hypothesized that there is functional redundancy of Oxt neurons in the AC. Neurons expressing Trh are distributed in the AC (Simerly et al., 1986), and the orthopaedia-expressing developmental progenitor cells are the same for Oxt and Trh neurons (Acampora et al., 1999). Moreover, Trh receptor (*Trhr*) was one of the transcripts upregulated in the dorsolateral MPOA of parenting mice in our previous DNA microarray study (Kuroda et al., 2007, 2008). These data prompted us to examine the maternal and alloparental behaviors of *Oxt-Trh* DKO mice (Fig. 3).

*Oxt-Trh* DKO mice were born at a Mendelian ratio, and appeared healthy and normal as their littermates with other genotypes, with a minor retardation of body growth of *Trh* KO mice (Yamada et al., 1997). The majority of DKO virgin females also showed normal allomaternal behaviors (Fig. 3*A*). DKO female mice mated and became pregnant similarly to their non-KO (heterozygous or wild-type) littermates (Fig. 3*B*). However, a significant proportion (18 out of 35, 51.4%) of *Oxt* KO mothers showed severe delay of parturition, such that the labor continued for >24 h after the delivery of the first pup, there was a dead pup found in the vaginal entry, or the maternal health deteriorated visibly, leading to spontaneous maternal death or euthanasia for the animal’s welfare (5 out of 35, 14.3%; Fig. 3*C*). The delayed-labor phenotype of *Oxt* KO mothers was not found in the initial studies in the mixed background (Nishimori et al., 1996; Young et al., 1996) but was suggested in a later study (Roizen et al., 2007), and significant in the present study in the C57BL/6 genetic background. These mothers that experienced abnormal labor were excluded from further analyses for maternal behaviors. The rate of abnormal labor was not significantly affected by the *Trh* genotype.

The home-cage postpartum maternal behaviors of *Oxt* KO and DKO mothers, including nest building, placentophagia, and pup grouping, were indistinguishable from those of their wild-type or heterozygous littermates (Fig. 3*D–F*). However, the pups born to *Oxt* KO mothers were weak, devoid of milk, and died within 2 d with 100% genetic penetrance (Fig. 3*G*; the three mothers with pups surviving through PPD1 lost them by PPD2), in concordance with previous literature (Nishimori et al., 1996; Young et al., 1996). At this time, we did not formally examine actual suckling behaviors; yet, the elongated nipples were found in all four *Oxt* KO mothers examined, with no clear milk bands observed in their litters, suggesting that the nursing behaviors *per se* were performed by *Oxt* KO mothers. Among six *Oxt* ^+/^ mothers examined, five mothers had elongated nipples, and four of them had the pups with a clear milk band. One *Oxt* heterozygous mother which did not have clearly elongated nipples had only one pup survive on the day of delivery.

Because unfed or unhealthy pups do not properly induce maternal retrieval, and because mouse mothers show maternal behaviors toward donor pups as well as their own pups, we performed pup retrieval assays with fed donor pups for all the subject mothers for consistency (see Kuroda et al., 2011). The *Oxt* KO mothers with or without *Trh* KO exhibited normal pup retrieval toward donor pups (Fig. 3*H*).

We next examined pup-directed behaviors in *Oxt*-*Trh* DKO male mice. DKO virgin and postmating (separated from females after copulation within a day) males performed infanticide as much as their littermates with other genotypes (Fig. 3*I*,*J*). After cohabitation with a female for 7 d, the proportion of males that successfully fertilized females was not significantly different among the genotypes (Fig. 3*K*). At the time of the females’ deliveries, no differences in pup-directed behaviors were found between DKO and their littermates (Fig. 3*L*). As previously reported, C57BL/6 fathers need to stay with the pregnant mate female until late gestation to become mostly paternal (Tachikawa et al., 2013). Two *Oxt* KO males stayed with their pregnant mates until late gestation, as well as three males that stayed with their mates until delivery, turned paternal. Overall, these results indicate no abnormalities in pup-directed behaviors in DKO male and female mice.

### Pup-directed behaviors of *Oxt*-*Trh*-*Avpr1a*-*Avpr1b* QKO female mice

Oxt’s role in pup-directed behaviors might be obscured by interactions with the Avp system, possibly at the receptor level. Two kinds of Avp receptors, Avpr1a and Avpr1b, are expressed in the brain (Koshimizu et al., 2012). Therefore, we created *Oxt*-*Trh*-*Avpr1a*-*Avpr1b* QKO mice, after crossing each line into the C57BL/6 genetic background at least four times.

During the breeding procedure, QKO male and female mice were born at a mendelian ratio, and appeared healthy. Moreover, they were grossly as fertile as their littermates with other genotypes, although we did not specifically examine their sexual behaviors in detail. The complete lack of milk transfer to the pups and the modest labor delay in *Oxt* KO mothers were consistently found throughout this study as shown in Figure 3. To focus on maternal behaviors, we excluded from further analyses the mothers that experienced a severely disturbed labor.

The breeding procedure was inevitably complicated and laborious, because there are 3̂4 (81) genotypes for the four gene loci. In this circumstance, testing all these genotypes separately was not practically feasible. Therefore, we categorized the genotypes into the following groups as in the Figure 4 one comparison between QKO versus all the other genotypes combined, and four comparisons for each gene locus as heterozygous versus KO (e.g., QKO vs *Oxt*^−/−^; *Trh*^−/−^; *Avpr1a*^−/−^; *Avpr1b^−/+^*: *QKO* vs *Oxt*^−/−^; *Trh*^−/−^; *Avpr1a^−/+^*; *Avpr1b*^−/−^, etc.).

Pup exposure assays were performed on four consecutive days in virgin females, on the day of delivery in the postpartum mothers and 13 d after the separation of pups at PPD1 (postisolation; Fig. 4*A*). In essence, QKO females showed grossly indistinguishable nurturing behaviors, including latencies for first sniffing, first and last pup retrieval, and durations of crouching over pups, licking, and nest building (Fig. 4*B–G*). The consistent changes of pup-directed behaviors between genotypes in Figures 4, 5 were the increased licking behavior in *Avpr1a* KO (thus also QKO) mothers at PPD0. Anecdotally, we noted a mild tendency of polyurea by the soiled bedding of *Avpr1a* KO mice, although normal basal urine output was reported for *Avpr1a* KO in a previous report in a genetically-mixed background (Koshimizu et al., 2006). The possible craving for water in *Avpr1a* KO and QKO postpartum mothers could have caused increased anogenital licking, and compensatory decrease of the crouching behavior. Other differences among genotypes were sporadic and not consistent (Fig. 5), suggesting the scarcity of robust deficits in maternal or allomaternal care of the QKO mutant females.

### Pup-directed behaviors of *Oxtr*-*Avpr1a*-*Avpr1b* TKO and *Oxt*-*Trh*-*Avpr1a*-*Avpr1b* QKO female mice

When the *Oxtr* KO mouse line (Takayanagi et al., 2005) became available to us, we created *Oxtr*-*Avpr1a*-*Avpr1b* TKO mice, after crossing each line into C57BL/6 genetic background at least four times, aiming at complete abolition of the Oxt-Avp system in the brain. First, we have examined whether the lack of *Oxtr* or *Avpr* genes affected the expression of Oxt or Avp though compensatory mechanisms, as suggested previously (Vaidyanathan and Hammock, 2020). In our experimental conditions, no significant differences of the expression levels of Oxt and Avp between *Oxtr* KO and heterozygous females were detected in the AC, SO, or PVH (Fig. 6).

As in *Oxt* KO mothers, complete lack of milk transfer to the pups (Takayanagi et al., 2005) and the mild labor delay in *Oxtr* KO mothers were also observed throughout this study. Again, we examined allomaternal and postpartum maternal behaviors in TKO females, excluding the mothers with a severely disturbed labor.

Similar to the results from QKO, we did not find any robust defects in maternal and allomaternal care in TKO females. The only consistent statistical significance (Fig. 4) was found in the increased licking duration in *Avpr1a* KO and TKO PPD0 females. Nipple elongation, indicating suckling, was found in all nine *Oxtr* KO mothers examined on PPD0, and no clear milk band was observed in the pups’ stomachs. In particular, pup-retrieval latencies, the reliably measurable index for parental motivation in mice (Kuroda et al., 2011), did not differ among genotypes for the first, second (data not shown), or the third pup (Figs. 4, 5) in either QKO, TKO or any single-locus KO females, indicating that there were no gross defects in pup retrieval in *Oxt*, *Oxtr*, *Avpr1a*, *Avpr1b*, *Trh* single KOs or in TKO or QKO females.

### Pup-directed behaviors in TKO male mice

Next, we examined the pup-directed behaviors of male mice, focusing on TKO mutant lines. In virgin *Oxtr* KO males, a significant increase of parental behavior in virgin males and decrease of infanticide in fathers on PPD1 were observed (Fig. 7*F*). The frequency of infanticide is affected by various environmental stimuli and internal factors (Parmigiani and vom Saal, 1994), and it is possible that an anxiolytic effect of Oxt may cause this difference. Paternal behavior at two different timepoints, however, was not significantly affected by any of the single KOs or by the TKO (Fig. 7*B–F*), as seen in our female mice.

### Confirmation of the results in another *Avpr1a* and *Avpr1b* mutant strains

The paucity of parental care-related phenotypes in the genetic mutants for the Oxt-Avp system so far identified was surprising. One concern about the genetic mutant line of *Avpr1a* used in the Figures 3-7 was that it was not a null mutation, but an insertion mutation, and there was no obvious phenotype. This means that we have to rely solely on the PCR genotyping to segregate the mutant from the wild-type allele, unlike the *Oxt* and *Oxtr* mutants where lack of milk is 100% observable in mutant mothers. We therefore obtained another targeted-mutant line of *Avpr1a* which has been confirmed for the phenotype in the cardiovascular system (Egashira et al., 2004; Koshimizu et al., 2006), along with the *Avpr1b* mutant line created in the same laboratory (Tanoue et al., 2004), and bred for the new TKO mice (i.e., the same mutant line for *Oxtr*, and independent lines for *Avpr1a* and *1b* from Figs. 5, 7), termed HIR.

Examination of virgin female, postpartum, virgin male, and paternal HIR TKO mice (Fig. 8) did not show any significant effects of any genotype on any pup-directed behaviors, except for the increased pup licking in one of three timepoints each in virgin and postpartum females, and decreased nest building behavior in one and two of the three timepoints each in virgin and postpartum females in the standard pup-exposure assay (Fig. 8*A*,*B*).

### Parental care of *Oxtr* KO is susceptible to physical stress

So far, our experiments did not elucidate the significant role of Oxt-Avp system in parental care, even in combination with mutations of 8 related genetic loci, indicating that the genetic redundancy among these genes was unlikely the cause of unsuccessful detection of phenotypes in pup-directed behaviors. However, it was still possible that the role of Oxt-Avp system in parental nurturing was not readily detectable in the minimal-stress laboratory environment. Actually, parental nurturing is always in trade-off with other drives, such as hunger and self-security (Li et al., 2019; Yoshihara et al., 2021), but these competing needs do not occur in laboratory environments. Therefore, we next examined the parental care in more stressful conditions, and found a significant decrease of nurturing behaviors if the virgin females were tested right after 30 min of restraint stress (Fig. 9; Extended Data Fig. 9-1). Three of the *Oxtr* KO virgin females exhibited infanticide toward donor pups after the restraint stress (Fig. 9*A*), although this effect did not reach statistical significance. The *Oxtr* KO virgin females also showed decreased pup retrieval, and thus the total nesting time summed for all the pups was reduced (Fig. 9*D*,*E*) in the pup-exposure assay performed right after restraint stress. On the other hand, this stress vulnerability caused by *Oxtr* KO was abolished by additional *Avpr1b* KO (Fig. 9*E*). Rather opposite effects of Oxtr and Avpr1b were found on nurturing behavior under stress. Such distinct roles of Oxtr and Avpr1b has been demonstrated in fear control in the central amygdala (Huber et al., 2005) and with the Bruce effect (Wersinger et al., 2008), suggesting the importance of fine balance of neurohypophysial hormones on behavioral outcome in these complex contexts.

### *Oxtr* KO females exhibit normal stress reactivity in nonmaternal context

To determine whether the effect of *Oxtr* KO in disturbing alloparental care is secondary to the well-known general anxiolytic effect of Oxt or not, the general stress sensitivities of the mutants were examined. Plasma cortisol levels after restraint stress, or the behavioral performances in the OF and EPM were not altered in *Oxtr* KO females (Fig. 10*A–C*). On the other hand, there was a significant increase of general anxiety in *Avpr1b* KO females, as evidenced consistently by the three measurements (Fig. 10*A–C*). This anxiogenic effect of *Avpr1b* was limited to females and not found in males, except for one measurement on the EPM (Fig. 10*D–F*). These results suggest that while the mild increase of alloparental nurturing after restraint stress in *Avpr1b* KO may be secondary to the general stress resistance, the decreased alloparenting after stress in *Oxtr* KO is rather specific to a pup-nurturing context.

## Discussion

This study has shown that the genetic mutation in *Oxtr* causes deficits in alloparental nurturing specifically under a stressful condition. This finding is in harmony with the previous literature, which unequivocally demonstrated anti-stress and anxiolytic effects of Oxt in various conditions, including in a semi-natural environment (Ragnauth et al., 2005; Brunton and Russell, 2008; Neumann, 2008; Yoshida et al., 2009; Viviani et al., 2011; Knobloch et al., 2012; Sabihi et al., 2014; Menon et al., 2018). Environmental stress and risk factors should be inevitable in the feral life, thus this role of Oxt should be important for maintaining motivation to nurture. The differences between our findings with *Oxtr* KO mice and those of Takayanagi et al. (2005) may have been because of the different stress levels involved in pup exposure assays (for details, see Yoshihara et al., 2017).

We also observed the modest deficits in parturition by either the *Oxt* or *Oxtr* mutation, a phenotype that was not observed in the original KO studies (Nishimori et al., 1996; Young et al., 1996; Gross et al., 1998; Takayanagi et al., 2005). However, this phenotype was observed partly in later studies (Roizen et al., 2007; Yoshida et al., 2019), possibly because of the difference in genetic backgrounds used. On the other hand, the complete lack of milk ejection despite vigorous suckling by starving pups was confirmed as originally reported (Nishimori et al., 1996; Young et al., 1996; Gross et al., 1998; Takayanagi et al., 2005).

Except for these phenotypes, however, this study has revealed the relative paucity of effects of *Oxt-Avp* genetic targeting on pup-directed behaviors in laboratory mice. In particular, we have found almost no robust facilitatory role of Oxt on maternal or allomaternal nurturing behaviors under our standard laboratory condition. And the TKO and QKO phenotypes indicate that the normal parental care is not because of the compensation for the congenital lack of Oxt or Oxtr by Avp or Trh molecular signaling. Of course, the compensation may occur via non-Avpr1a, non-Avpr1b, non-Trh mechanism. In contrast to the inability to compensate for the deficit in milk ejection because of the lack of *Oxt* or *Oxtr*, these data provide no evidence for Oxt-Avp-Trh’s role in parental care, at least after chronic loss. Whether acute loss of one, two or all three neuropeptide signaling systems would affect parental care can be tested by acute molecular knock-down through AAV-mediated RNA interference, for example.

This study has several limitations other than the use of unnaturally, relatively stress-free laboratory conditions. For example, unlike laboratory mouse strains, virgin female wild mice are often not spontaneously maternal, and even unresponsive or infanticidal toward unfamiliar pups (McCarthy and vom Saal, 1985; Soroker and Terkel, 1988; Chalfin et al., 2014). Moreover, domesticated mice show differences in Oxt and Avp expression in brain regions known to regulate social behavior and emotion (Ruan and Zhang, 2016). Therefore, inbreeding and domestication may have rendered lab mice even less dependent on the facilitatory effects of Oxt for the initiation of maternal behavior in inexperienced (first-time) mothers under standard laboratory conditions.

Second, the studied functions of Oxt neurons may be mediated by co-expressing neurotransmitters or neuropeptides, such as glutamate (Xu et al., 2020), corticotropin-releasing factor, cholecystokinin and dynorphin (Levin and Sawchenko, 1993). If this is the case, while Oxt molecules are not important, Oxt neurons may be important, and the function of Oxt neurons on pup-directed behaviors should be detected by manipulation of Oxt expressing neuronal activity by using AAV-mediated tetanus toxin or Gi-DREADD, for example, as performed in previous work (Tang et al., 2020).

Lastly, Oxt function may be more visible in nursing behavior and/or in the later lactation period. We observed nipple elongation, evidence for nursing on the day of delivery, consistent with the previous finding with a forebrain-specific *Oxtr* KO (Macbeth et al., 2010). However, we did not formally exclude other possible problems in nursing-related behaviors. In particular, the Oxt neurons could be involved in the phenomenon called “Pavlovian milk conditioning.” It has been known anecdotally that experienced lactating human and dairy animal mothers let down milk with conditional stimuli, such as a infant cry or smell, and bells signaling milking time in a dairy farm, all before physical suckling (Grosvenor and Mena, 1972; McNeilly et al., 1983; Fuchs et al., 1987; Tancin et al., 2001; Domjan, 2005). Once this conditioning is formed, the mothers may have milk letdown just by approaching and receiving infant sensory cues, or in human cases, just to think about the infants, before actual suckling by the infants, thus facilitating milk transfer to the infants. We recently identified a key player in maternal care, Calcr-expressing neurons in the cMPOA and the AC (Yoshihara et al., 2021). Calcr-expressing neurons are significantly activated during and required for maternal and allomaternal care. The close spatial relationship of Oxt neurons and Calcr neurons in the AC suggests that simultaneous firing of these neurons should occur repeatedly in the AC during maternal care, and may form the synaptic connections between these neuronal populations, which may be responsible for the Pavlovian milk conditioning. If this is the case, it may best explain why there is a striking co-distribution of the abundant activated nonoxytocinergic neurons among Oxt neurons in the AC and Oxt-fibers in the MPOA.

Even positive reports for Oxt’s facilitation of parental care show relatively mild effects on specific components of parental care (Pedersen et al., 1994; Takayanagi et al., 2005; Marlin et al., 2015; Carcea et al., 2021), rather than abolishing many aspects of complex parental behaviors. Further investigations are needed to examine these possibilities and determine the exact role of Oxt system in pup-directed behaviors. However, at least the present findings provide a caution for the pervasive, yet not-well substantiated, claim of an indispensable role of Oxt in all kinds of parental care.

## Synthesis

Reviewing Editor: Erica Glasper, The Ohio State University Decisions are customarily a result of the Reviewing Editor and the peer reviewers coming together and discussing their recommendations until a consensus is reached. When revisions are invited, a fact-based synthesis statement explaining their decision and outlining what is needed to prepare a revision will be listed below. The following reviewer(s) agreed to reveal their identity: Danielle Stolzenberg. Note: If this manuscript was transferred from JNeurosci and a decision was made to accept the manuscript without peer review, a brief statement to this effect will instead be what is listed below.

Summary: The authors of this manuscript conducted an extremely thorough pre-clinical investigation to determine the role of oxytocin (OT), vasopressin (AVP), and thyrotropin releasing factor (TRF) in parental behaviors in male and female mice with and without parental history. This work is necessary to understand the potential cross-talk between the aforementioned neuropeptides and hormones that may explain the regulation of pup-directed care. Seven different knockout strains were used to understand the critical role of these peptides and their receptors on parental behavior expression. While OT has been emphasized as critical for maternal care, other work demonstrates that OT or OT receptor deficient mice display normal maternal behavior.

Concerns: The methods and findings described in this manuscript were thoroughly discussed, however, the organization of the manuscript is confusing in certain places (see below) that makes the presented data difficult to follow at times. Given this, the manuscript failed to synthesize its major findings into a summary message; this is apparent in the Abstract also. Overall, the manuscript was well-written, however, clarity could be enhanced in specific places (see below). Where appropriate, reviewer’s specific comments were copied and pasted into this synthesis. They can be found in italicized text.

Major Concerns:

1. A major concern is the use of the domesticated lab mouse as a model. While domesticated virgin mice exhibit pup care, wild mice do not (Chalfin et al. 2014, Nature Communications). Moreover, domesticated mice show differences in oxytocin and vasopressin expression in brain regions known to regulate social behavior and emotion (Ruan & Zhang, 2016, Anatomical Science International). The choice of the domesticated lab mouse therefore selects for spontaneous parental responsivity that may or may not be mediated by the same neural mechanisms exhibited in wild mice. The authors are encouraged to discuss their null findings in the light of this fact.

2. Clarification of genetic line usage: The paper had to be read several times to understand what genetic lines were used when and for what reason. The authors are encouraged to create a schematic table, which should include 1) each genotype used, 2) indicate which mice were considered “wildtypes” (with the symbol), and 3) include a column that lists a brief (∼1 sentence) rationale for the use of each line.

3. The N sizes in the paper are not clearly identified. This information should be clearly presented and described in the figure captions, figure legends, or the main text. The authors should ensure that the number of mice/group are in agreement within the text and within the figures (e.g., Table I does not appear to include the double KO (avp1ar/trh) that is illustrated in Figure 4A very first column.) It appears that N=1 for this group; this should be clarified. Additionally, please define DSTX and DRS in Table 2.

4. An additional recommendation is the use of a timeline. Again, while the methods are thorough, there are places where the timeline is unclear (e.g., Figure 4B seems to indicate that the same mice were tested as virgins and postpartum? If this is not the case (which I am assuming it’s not), then the use of line graphs is not appropriate. It seems like post-isolation and postpartum groups are the same, but again I didn’t find a description of this.)

5. The graphs within the figures could be re-organized to improve readability. For example, in figure 3 the graphs should be arranged so that the legends match the graphs for a given block. This will prevent the reader from having to switch back and forth between the legends for each graph. Another concern is the use of the same legend for a number of graphs. For example, why use the same legends for A, D, E, and G? Can’t those graphs be placed next to each other? More clarity could also be gained by changing the names of the graph titles. For example, in “A” while it useful to know that the data represent virgin female mice, I have to go through the legend to understand that you are referring to maternal behavior responses in virgin female mice. Perhaps the behavior graphs could be organized into a block that indicates the behavioral assay and the group of mice being tested as a subheading.

6. The discussion section could be improved by discussing the negative findings of the study instead of stating that the data are not included (e.g., calcitonin receptor discussion).

Minor Concerns:

1. In the introduction, it may be appropriate to remove the reference relating to human given that a recent attempt to replicate these findings failed.

2. In the methods sections, please clarify which mice were sacrificed via decapitation for trunk blood and hormone measurement versus those mice that were perfused for brain histology. Specifically, please indicate when the perfusion occurred relative to testing and how these cohorts overlapped and/or were different. As mentioned in Major Comment #3, the use of timelines may facilitate the need for clarity.

3. Additionally in the methods section, there are dissimilarities between strain-specific testing paradigms. This should be clarified.

4. Page 9, line 230, “par” should be “per"

5. Page 9, line 241, “par” should be “per"

Readers may benefit from an explanation of how NPI relates to OT.

6. Lines 365-366: Please discuss whether the findings that c-Fos-ir was limited to non-oxytocinergic neurons contrast with the recent findings of Carcea et al. (2021) on oxytocin and female alloparental care.

7. Line 472: Please indicate whether Avpr1aKO animals display decreased licking in one experiment and increased licking in another. If the answer is yes, please provide an explanation.

8. In Figure 2, the circle-headed arrow is difficult to detect as it looks similar to c-Fos-ir. Please change the color of this arrowhead.

9. In the discussion, the authors are encouraged to include references that support the GABA argument. Specifically, is there a reference that can be included showing OXT’s ability to bind/affect GABA receptors?
